# The Human Centrosomal Protein CCDC146 Binds *Chlamydia trachomatis* Inclusion Membrane Protein CT288 and Is Recruited to the Periphery of the *Chlamydia*-Containing Vacuole

**DOI:** 10.3389/fcimb.2018.00254

**Published:** 2018-07-26

**Authors:** Filipe Almeida, Maria P. Luís, Inês Serrano Pereira, Sara V. Pais, Luís Jaime Mota

**Affiliations:** ^1^Research Unit on Applied Molecular Biosciences (UCIBIO) – REQUIMTE, Departamento de Ciências da Vida, Faculdade de Ciências e Tecnologia, Universidade Nova de Lisboa, Costa da Caparica, Portugal; ^2^Instituto de Tecnologia Química e Biológica António Xavier, Universidade Nova de Lisboa, Oeiras, Portugal

**Keywords:** host-pathogen interactions, *Chlamydia trachomatis*, type III secretion, Inc proteins, centrosome

## Abstract

*Chlamydia trachomatis* is an obligate intracellular human pathogen causing mainly ocular and genital infections of significant clinical and public health impact. *C. trachomatis* multiplies intracellularly in a membrane bound vacuole, known as inclusion. Both extracellularly and from within the inclusion, *C. trachomatis* uses a type III secretion system to deliver several effector proteins into the cytoplasm of host cells. A large proportion of these effectors, the inclusion membrane (Inc) proteins, are exposed to the host cell cytosol but possess a characteristic hydrophobic domain mediating their insertion in the inclusion membrane. By yeast two-hybrid, we found that *C. trachomatis* Inc CT288 interacts with the human centrosomal protein CCDC146 (coiled-coil domain-containing protein 146). The interaction was also detected by co-immunoprecipitation in mammalian cells either ectopically expressing CCDC146 and CT288 or ectopically expressing CCDC146 and infected by a *C. trachomatis* strain expressing epitope-tagged and inclusion membrane-localized CT288. In uninfected mammalian cells, ectopically expressed full-length CCDC146 (955 amino acid residues) localized at the centrosome; but in cells infected by wild-type *C. trachomatis*, its centrosomal localization was less evident and CCDC146 accumulated around the inclusion. Recruitment of CCDC146 to the inclusion periphery did not require intact host Golgi, microtubules or microfilaments, but was dependent on chlamydial protein synthesis. Full-length CCDC146 also accumulated at the periphery of the inclusion in cells infected by a *C. trachomatis ct288* mutant; however, a C-terminal fragment of CCDC146 (residues 692–955), which interacts with CT288, showed differences in localization at the periphery of the inclusion in cells infected by wild-type or *ct288* mutant *C. trachomatis*. This suggests a model in which chlamydial proteins other than CT288 recruit CCDC146 to the periphery of the inclusion, where the CT288-CCDC146 interaction might contribute to modulate the function of this host protein.

## Introduction

*Chlamydiae* are a large group of obligate intracellular bacteria, including human and animal pathogens, and symbionts of free-living amoebae. Among *Chlamydiae, C. trachomatis* is an important human pathogen causing ocular and genital infections. *C. trachomatis* ocular strains (serovars A–C) are the leading cause of infectious blindness (trachoma; Taylor et al., 2014), urogenital strains (serovars D–K) are the most common cause of bacterially sexually transmitted diseases worldwide, and lymphogranuloma venereum (LGV) strains (serovars L1–L3) cause invasive urogenital or anorectal infection (O'Connell and Ferone, 2016).

As all *Chlamydiae, C. trachomatis* undergoes a developmental cycle involving the inter-conversion between an infectious form, the elementary body (EB), and a non-infectious form, the reticulate body (RB). Attachment of EBs to host cells leads to bacterial invasion with the formation of a *Chlamydia*-containing vacuole known as inclusion. Early after uptake, EBs start differentiating into RBs and the inclusion segregates from normal endosomal maturation. Through the activity of cytoplasmic dynein, the nascent inclusion migrates along microtubules toward a centrosomal region. RBs then divide, while membrane and nutrients are routed to the inclusion by interaction with host cell vesicular and non-vesicular transport pathways. During the cycle, the inclusion remains near the host centrosome and nucleus. Eventually, most RBs differentiate back into EBs, which exit the host cell and can infect neighboring cells [reviewed by (Elwell et al., 2016) and references therein].

Throughout the developmental cycle, *Chlamydiae* manipulate host cells by using a type III secretion (T3S) system to deliver several effector proteins into host cells (Mueller et al., 2014). Chlamydial T3S substrates include inclusion membrane (Inc) proteins (Subtil et al., 2001), a family of *Chlamydiae*-unique proteins characterized by a bilobed hydrophobic motif thought to mediate their insertion into the inclusion membrane (Rockey et al., 2002). Bioinformatics indicates that *C. trachomatis* encodes about 60 Inc proteins (Bannantine et al., 2000; Dehoux et al., 2011; Lutter et al., 2012), and 37 of them have been experimentally detected in the inclusion membrane (Weber et al., 2015).

Considering that Inc proteins reside at the interface of the inclusion and the host cell cytosol, it is not surprising that many Inc proteins have been shown to play a role in mediating *Chlamydia*-host cell interactions. For example, *C. trachomatis* CT005/IncV tethers the inclusion to host the endoplasmic reticulum (Stanhope et al., 2017); CT101/MrcA and CT228 regulate the process of *C. trachomatis* release from host cells (Lutter et al., 2013; Nguyen et al., 2018); CT115/IncD interferes with host non-vesicular transport (Derré et al., 2011); CT116/IncE (Mirrashidi et al., 2015) and CT119/IncA (Delevoye et al., 2008) modulate host cell vesicular trafficking; CT223/IPAM and CT813/InaC are involved in the manipulation of host cell microtubules (Dumoux et al., 2015; Wesolowski et al., 2017); CT813/InaC also promotes actin assembly and Golgi redistribution around the inclusion (Kokes et al., 2015); and CT850 binds dynein light chain and thereby mediates transport of the inclusion toward the centrosome (Mital et al., 2015). In addition, CT229/CpoS inhibits host cell death in *C. trachomatis*-infected cells (Sixt et al., 2017; Weber et al., 2017). This could be related to its potential capacity to modulate host cell vesicular trafficking (Rzomp et al., 2006; Mirrashidi et al., 2015; Sixt et al., 2017), or to its role in the stability of the inclusion membrane (Weber et al., 2017). CT233/IncC and CT383 are also essential for the stability of the inclusion membrane (Weber et al., 2017). Together with the observation that some Inc proteins form homo- and heterooligomers (Gauliard et al., 2015), this is in line with the idea that some Inc proteins could have mainly a structural role (Mital et al., 2013; Moore and Ouellette, 2014). Finally, a large proteomics study identified interactions of 38 Incs with several host proteins (Mirrashidi et al., 2015). However, the function of many Inc proteins remains undefined.

In this work, we identified the host centrosomal protein CCDC146 (coiled-coil domain-containing protein 146) (Firat-Karalar et al., 2014) as an interacting partner of *C. trachomatis* Inc CT288 and found that in infected cells CCDC146 is recruited to the periphery of the inclusion in a CT288-independent fashion. However, CT288 might modulate the function of CCDC146 at the inclusion.

## Materials and methods

### Mammalian cell lines

HeLa 229, HEK293T and Vero cells (all from the European Collection of Cell Culture; ECACC) were maintained in high-glucose Dulbecco's modified Eagle Medium (DMEM; Thermo Fisher Scientific) supplemented with heat-inactivated 10% (v/v) fetal bovine serum (FBS; Thermo Fisher Scientific) at 37°C in a humidified atmosphere of 5% (v/v) CO_2_. Cells were checked for *Mycoplasma* by conventional PCR either using the Venor® GeM Advance kit (Minerva Biolabs) or as described (Uphoff and Drexler, 2011).

### Bacterial strains and growth conditions

*Escherichia coli* TOP10 (Thermo Fisher Scientific) or NEB® 10β (New England Biolabs) were used for construction and purification of plasmids, and *E. coli* ER2925 (New England Biolabs) to amplify and purify plasmids for transformation of *C. trachomatis*. *E. coli* strains were routinely grown in liquid or solid lysogeny broth (LB) medium with the appropriate antibiotics and supplements. Plasmids were introduced into *E. coli* by electroporation.

*Chlamydia trachomatis* LGV serovar L2 strain 434/Bu (L2/434; from ATCC) was propagated in HeLa 229 cells using standard techniques (Scidmore, 2005). Throughout this work we used the nomenclature of the annotated *C. trachomatis* D/UW3 strain (Stephens et al., 1998). Transformation of *C. trachomatis*, first reported by the laboratory of Ian Clarke (Wang et al., 2011), was done essentially as described (Agaisse and Derre, 2013). The optimal antibiotic concentrations to select transformants were 1 U/ml of penicillin G, or 250 μg/ml of spectinomycin. Once established, the transformed strains were cultured in the presence of 10 U/ml of penicillin, or 500 μg/ml of spectinomycin, and plaque purified using Vero cells, as described (Nguyen and Valdivia, 2013). Infection of mammalian cells by *C. trachomatis* and quantification of infection progeny was done as previously described (da Cunha et al., 2017).

### DNA manipulation, plasmids, and primers

The plasmids used in this work, their main characteristics and construction details, are described in Table S1. The DNA primers used in their construction are listed in Table S2. Plasmids were constructed and purified using standard molecular biology procedures with proof-reading Phusion DNA polymerase (Thermo Fisher Scientific), restriction enzymes (Thermo Fisher Scientific), T4 DNA Ligase (Thermo Fisher Scientific), DreamTaq DNA polymerase (Thermo Fisher Scientific), DNA clean and concentrator™-5 kit and Zymoclean™ gel DNA recovery kit (Zymo Research), and GeneElute Plasmid Miniprep kit (Sigma Aldrich) or NZYMidiprep kit (NZYtech), according to the instructions of the manufacturers. The backbone plasmids used in this work were: pGADT7 (Clontech) and pGBKT7 (Clontech), used for yeast two-hybrid assays; pEGFP-C1 (Clontech), and pEF6/*myc*-His C (Thermo Fisher Scientific), used to generate mammalian transfection plasmids; and pSVP247 (da Cunha et al., 2017), a derivative of p2TK2—SW2 (Agaisse and Derre, 2013), used to generate a *C. trachomatis* expression plasmid bearing CT288 with a double hemagglutinin epitope tag (2HA) at its C-terminus (CT288-2HA). The accuracy of the nucleotide sequence of all the inserts in the constructed plasmids was confirmed by DNA sequencing.

### Construction of a *C. trachomatis ct288::aadA* mutant strain

A *C. trachomatis ct288::aadA* mutant was generated using group II intron-based insertional mutagenesis, as previously described (Johnson and Fisher, 2013; Key and Fisher, 2017). Briefly, intron-insertion sites in the *C. trachomatis* L2/434 *ctl0540* gene (ortolog of *ct288* in *C. trachomatis* strain D/UW3) were identified using the TargeTron algorithm (Sigma). Then, the intron in pDFTT3 aadA (Table S1; Key and Fisher, 2017) was retargeted for *ctl0540* using standard molecular biology procedures and the DNA primers listed in Table S2. The *ctl0540* mutator plasmid (pML2; Table S1) was then used to transform *C. trachomatis* L2/434, as described above (in bacterial strains and growth conditions).

### Yeast two-hybrid (Y2H) screen

The Matchmaker® Gold Yeast Two-Hybrid System (Clontech) was used to screen of a pre-transformed Mate and Plate™ Library—normalized Universal Human HeLa cDNA library (Clontech), following the instructions of the manufacturer. In summary, *Saccharomyces cerevisiae* Y2HGold (Clontech) was transformed with plasmid pFA147, a derivative of pGBKT7 (Table S1) and mated with yeast strain Y187 (Clontech) carrying the HeLa cDNA library cloned into pGADT7. The two strains were mated and plated in lower stringency or double dropout (DDO) media (SD/–Leu/–Trp) supplemented with X-α-Galactosidase (X) and Aureobasidin A (A) (all from Clontech) and incubated 3–5 days at 28–30°C. The blue colonies grown on DDO/X/A media were patched into a higher stringency or quadruple dropout (QDO) media (SD/–Ade/–His/–Leu/–Trp) supplemented with X-α-Galactosidase and Aureobasidin A and incubated 3–5 days at 28–30°C. The blue colonies that grew in QDO/X/A were further analyzed for autonomous system activation, and for identification of the target protein, by sequencing the plasmid DNA (primers listed in Table S2). We analyzed 1.43 × 10^8^ clones, patched 60 blue colonies and recovered 41 from high stringency media for further analysis.

### Preparation of yeasts extracts

Protein extracts of the *S. cerevisiae* Y2HGold strains expressing Gal4 DNA binding domain fusion proteins were prepared by the trichloroacetic acid (TCA) method, as described in the supporting protocols of the Matchmaker® Gold Yeast Two-Hybrid System (Clontech).

### Transient transfection of mammalian cells

HeLa 229 and HEK293T cells were transfected with plasmid DNA by using the jetPEI reagent (Polyplus-Transfection) as detailed in the instructions of the manufacturer but using 250 ng of DNA per well of a 24-well tissue culture plate or 1,250 ng of DNA per well of a 6-well tissue culture plate. When seeding HEK293T cells, the tissue culture plates were previously coated with 0.001% (v/v) poly-L-lysine (Sigma Aldrich) in phosphate buffered saline (PBS). Cells were transfected immediately after infection with *C. trachomatis* and incubated at 37°C in a humidified atmosphere of 5% (v/v) CO_2_, prior to collection and immunoblotting, or fixation and immunolabeling.

### Preparation of cell lysates, fractionation and co-immunoprecipitation (co-IP)

For the co-IP, we used GFP-Trap kit (ChromoTek), according to the protocol of the manufacturer with minor adjustments. In experiments involving only co-transfection, 5 × 10^5^ HEK293T cells per well of a 6-well tissue culture plate, in a total of 2 wells per condition, were transfected with combinations of two plasmids, one encoding an EGFP fusion protein and the other encoding a HA-tagged protein. In experiments involving simultaneous infection and transfection, 5 × 10^5^ HEK293T cells per well of a 6-well tissue culture plate, in a total of 12 wells per condition, were infected with *C. trachomatis* transformed with pSVP255 (pCT288-2HA; Table S1) at a multiplicity of infection of 2, and transfected with plasmids encoding for EGFP, EGFP-CCDC146_692−955_, or EGFP-CCDC146_1−955_ (Table S1). In both cases, after 24 h of incubation at 37°C in a humidified atmosphere of 5% (v/v) CO_2_, the cells were collected by trypsinization, pelleted by centrifugation and washed with PBS. In the case of the simultaneous infection and transfection experiments, an additional crosslinking step was done. For that, the cell pellets were resuspended in 1 ml of paraformaldehyde 1% (w/v) in PBS per 1 × 10^7^ cells and incubated 7 min at room temperature. The cells were immediately centrifuged for 3 min at 1,800 × g at room temperature. Then, the pellets were washed twice with 500 μl 1.25 M glycine, and centrifugations of 3 min, 1,800 × g, room temperature. The remainder of the procedure was identical for co-transfection, and simultaneous infection and transfection experiments. The cell pellets were lysed in 200 μl ice-cold co-IP lysis buffer (20 mM Tris-HCl pH 7.5, 137 mM NaCl, 2 mM EDTA, 1% [v/v] NP40, freshly added protease inhibitors [Amresco] and phenylmethylsulfonyl fluoride [PMSF]) for 30 min on ice, and mixed by pipetting every 10 min. The lysates were centrifuged for 10 min, 17,000 × g, 4°C, and the supernatants were either used for analysis in SDS-PAGE (input of co-IP) or added to 800 μl of ice-cold GFP Trap buffer (10 mM Tris-HCl pH 7.5, 150 mM NaCl, 0.5 mM EDTA). Then, 750 μl of the diluted supernatant were added to previously washed GFP-Trap beads and incubated overnight at 4°C with end-over-end mixing. The beads were washed six times with GFP-Trap buffer with centrifugations of 2 min, 2,000 × g, 4°C. Finally, the pelleted beads (output of co-IP) and the input of co-IP (20 ul) were resuspended in 20 μl of SDS-PAGE buffer 2X (100 mM Tris-HCl, pH 6.8, 4.0% [w/v] SDS, 20% [v/v] glycerol, 0.2 M β-mercaptoethanol, 0.2% [w/v] bromophenol blue). The samples were incubated for 10 min at 100°C and the proteins were analyzed by immunoblotting.

### Antibodies and fluorescent dyes

For immunoblotting, mouse anti-*myc* antibody (Calbiochem) was used at 1:1,000, mouse anti-α-tubulin (Sigma-Aldrich) was used at 1:1,000, rat anti-HA 3F10 (Roche) was used at 1:1,000, goat anti-GFP (Sicgen) was used at 1:1,000, goat anti-MOMP (Abcam) was used at 1:1,000, and mouse anti-CT288 (a gift from Guangming Zhong; Li et al., 2008) was used at 1:1,000. Horseradish peroxidase-conjugated secondary antibody anti-mouse (GE Healthcare) was used at 1:10,000, anti-rat (Sigma-Aldrich) was used at 1:10,000, and anti-goat (Jackson ImmunoResearch Laboratories) was used at 1:10,000. For immunofluorescence, mouse anti-γ-tubulin antibody (Sigma-Aldrich) was used at 1:200, mouse anti-CT442 antibody (a gift from Guangming Zhong; Li et al., 2008) was used at 1:200, rat anti-HA 3F10 antibody (Roche) was used at 1:200, goat anti-GFP antibody (Sicgen) was used at 1:200, and goat anti-MOMP antibody (Abcam) was used at 1:200. Secondary antibody donkey anti-goat conjugated to cyanine 5 (Jackson ImmunoResearch Laboratories) was used at 1:200, anti-rat conjugated to rhodamine RedX (Jackson ImmunoResearch Laboratories) was used at 1:200, and goat anti-mouse AF568 (Invitrogen) was used at 1:200. 4′,6-diamidino-2-phenylindole (DAPI; Thermo Fisher Scientific) was used to stain DNA.

### Immunoblotting

To prepare total mammalian cell extracts, HeLa 229 cells were trypsinized, collected and centrifuged 5 min, 2,300 × g, 4°C. The pellet was washed twice with ice-cold PBS and resuspended in 50 μl of SDS-PAGE buffer 1X (50 mM Tris-HCl 50 mM, pH 6.8, 2.0% [w/v] SDS, 10% [v/v] glycerol, 0.1 M β-mercaptoethanol, 0.1% [w/v] bromophenol blue) and 1 μl benzonase (Novagen). The samples were incubated 10 min at 100°C. Total yeast cell extracts, total mammalian cell extracts, and fractionated co-IP samples were resolved in 12% SDS-PAGE, followed by immunobloting. Proteins in the gel were transferred onto nitrocellulose membranes (Bio-Rad) and blocked in 4% (w/v) dried skimmed milk diluted in PBS containing 0.1% (v/v) Tween-20. The membranes were probed with primary and horseradish peroxidase-conjugated secondary antibodies and generally detected using SuperSignal™ West Pico Chemiluminescent Substrate (Thermo Fisher Scientific), or SuperSignal™ West Femto Maximum Sensitivity Substrate (Thermo Fisher Scientific), in a Chemidoc XRS+ system (Bio-Rad) or by exposure to Amersham Hyperfilm ECL (GE Healthcare).

### Immunofluorescence microscopy

For immunofluorescence microscopy, cells were either fixed with 4% (w/v) paraformaldehyde for 15 min at room temperature or with methanol for 5 min at −20°C. Antibodies were diluted in PBS with 0.1% (v/v) Triton-X100 and 10% (v/v) horse serum. The cells were washed in PBS with 0.1% (v/v) Triton-X100 and incubated for 1 h with primary antibodies. The cells were washed again in PBS with 0.1% (v/v) Triton-X100 and incubated with appropriate secondary antibodies for another 1 h. The cells were washed again first in PBS with 0.1% (v/v) Triton-X100, then PBS, and finally in H_2_O. In the end, coverslips were mounted onto glass slides using Aqua-poly/Mount mounting medium (Polysciences). Samples were analyzed using widefield fluorescence microscopes (Leica DMRA2 or Zeiss AxioImager D2) or a confocal laser scanning microscope (Zeiss LSM 710 META). Unless otherwise indicated, images were obtained by confocal microscopy and processed using Zeiss LSM Image Browser and Adobe Photoshop software or Fiji (Schindelin et al., 2012).

### Drug treatments

To disrupt the host Golgi complex, microtubules, or microfilaments, *C. trachomatis*-infected cells were incubated with 1 μg/ml of Brefeldin A (BFA; Sigma; stock solution at 5 mg/ml in dimethyl sulfoxide [DMSO]), 1 μg/ml nocodazole (stock solution at 5 mg/ml in DMSO; Sigma), or 2 μM cytochalasin D (stock solution at 5 mg/ml in DMSO; Sigma), respectively. To inhibit bacterial protein synthesis, *C. trachomatis* infected cells were incubated with 200 μg/ml of chloramphenicol (Carl Roth; stock solution at 34 mg/ml in ethanol).

### Statistical analyses

Statistical analyses were done using GraphPad Prism, version 5.04 for Windows, GraphPad Software, San Diego California, USA (www.graphpad.com)]. Differences between data sets were considered significant if *P* < 0.05.

## Results

### *C. trachomatis* Inc protein CT288 binds the human centrosomal protein CCDC146 in a Y2H assay

**Figure 1:**
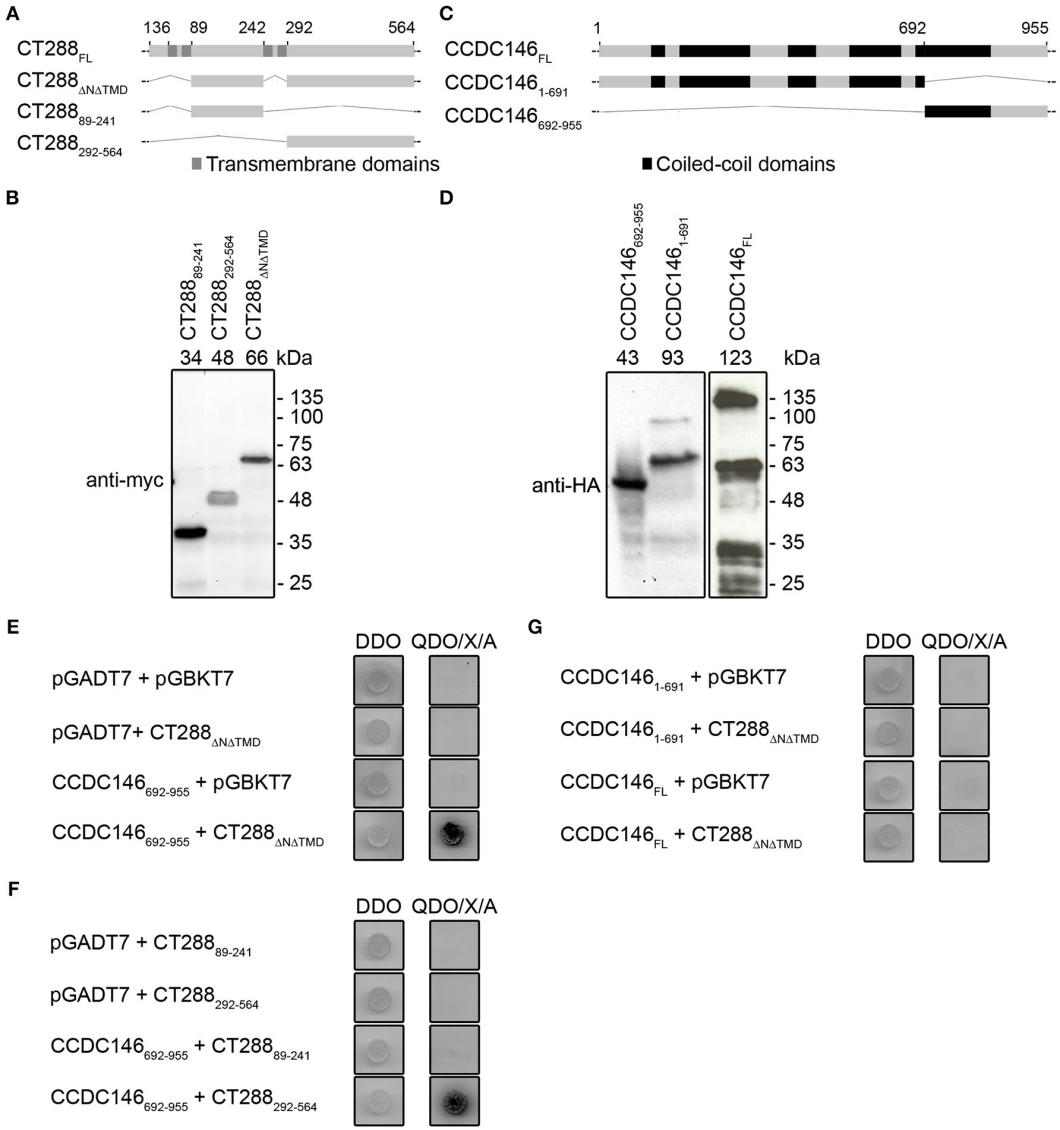
*Chlamydia trachomatis* Inc CT288 binds to human centrosomal protein CCDC146 by yeast two-hybrid (Y2H). **(A,C)** Schematic representation of CT288 and CCDC146 and of the fragments of these proteins used in Y2H assays. The predicted transmembrane domains of CT288 and coiled-coil domains of CCDC146 (as indicated in UniProt Q8IYE0; The UniProt, 2017) are highlighted. **(B,D)** Immunoblots of protein extracts of *S. cerevisiae* Y2HGold strains producing **(B)** myc-tagged fusions of CT288 fragments to the Gal4 DNA-binding domain or **(D)** HA-tagged fusions of CCDC146 to the Gal4 activation domain. The numbers above the blot indicate the predicted molecular mass of the corresponding fusion proteins. **(E)** Interaction between CT288_Δ*NΔTMD*_ and CCDC146_692−955_ by Y2H. **(F)** Interaction between CT288_292−564_ and CCDC146_692−955_ by Y2H, but not between CT288_89−241_ and CCDC146_692−955_. **(G)** CCDC146_FL_ and CCDC146_1−691_ do not bind CT288_Δ*NΔTMD*_ by Y2H. In **(E**–**G)**, DDO, double dropout media; QDO/X/A, quadruple dropout media supplemented with X-α-Galactosidase and Aureobasidin A (see Materials and Methods;) yeast growth as blue colonies (dark in the image) in high stringency QDO/X/A media indicates a protein-protein interaction. The empty pGADT7 and pGBKT7 plasmids were used as controls in the Y2H assays.

*Chlamydia trachomatis* Inc protein CTL0540 (herein CT288) from L2/434 strain is composed of 564 amino acid (aa) residues and encompasses bilobed hydrophobic motifs between residues 36–88, and 242–291 (Figure 1A). CT288 is different from most Inc proteins in that it contains more than one bilobed hydrophobic motif (Dehoux et al., 2011). To identify host cell interacting partners of CT288, we performed a Y2H screen using CT288 deleted of its first 35 aa residues and of its bilobed hydrophobic regions (CT288_Δ*NΔTMD*_; Figures 1A,B) as bait for proteins expressed from a human HeLa cell line cDNA library. After discarding false positives (i.e., autonomously activating the system or out-of-frame relative to the coding region of the Gal4 activation domain; GAL4AD), this resulted in the identification of 29 interacting protein fragments (Table S3). Among these CT288-interacting protein fragments, 9 (31%) were the C-terminal region (residues 692–955 or 721–955) of the 955 residues-long CCDC146 (Figure 1C and Table S3), which has been identified as a centrosomal protein (Firat-Karalar et al., 2014). Additional assays showed that CT288_Δ*NΔTMD*_ can bind CCDC146_692−955_ (Figures 1D,**E**). We next used Y2H to identify the region of CT288 involved in binding to CCDC146_692−955_. For this, Y2H plasmids encoding CT288_89−241_ and CT288_292−564_ were constructed (Figures 1A,B). Using these plasmids, we observed that CT288_292−564_, but not CT288_89−241_, binds to CCDC146_692−955_ (Figure 1F). We also tested if CT288_Δ*NΔTMD*_ could bind to full-length CCDC146 (CCDC146_FL_) and to the N-terminal region of CCDC146 (CCDC146_1−691_) by Y2H (Figures 1C,D). However, in this assay, we did not detect binding of CT288_Δ*NΔTMD*_ to either CCDC146_FL_ or CCDC146_1−691_ (Figure 1G).

As we previously showed that CT288 from *C. trachomatis* LGV strains contains 18 unique aa residues that are different from its orthologues in ocular and urogenital strains (Almeida et al., 2012), we tested if CT288 from *C. trachomatis* serovar C (ocular strain TW3) and from serovar E (urogenital strain BOUR) were able to bind CCDC146_692−955_. For this, plasmids encoding CT288_Δ*NΔTMD*_ from these strains were constructed (Figure S1A). Using these plasmids, we showed that CT288_Δ*NΔTMD*_ from *C. trachomatis* ocular and urogenital strains also binds CCDC146_692−955_ in a Y2H assay (Figure S1B).

Overall, these experiments showed that *C. trachomatis* Inc CT288 can bind the human centrosomal protein CCDC146 in a Y2H assay.

### CT288 and CCDC146 interact after their ectopic expression in human cells

**Figure 2:**
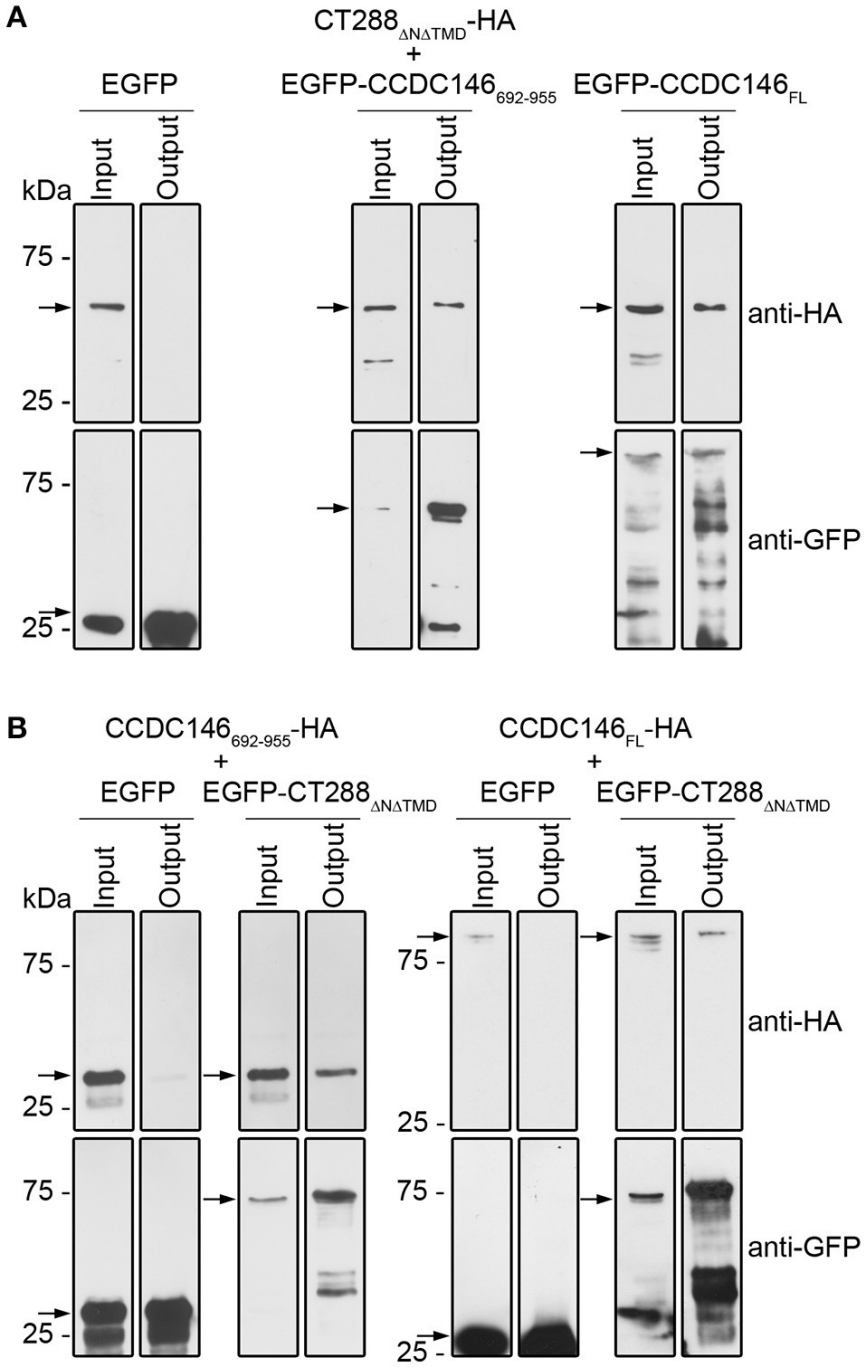
CT288 and CCDC146 interact after ectopic expression in mammalian cells. Plasmids encoding the indicated proteins were used to transfect HEK293T cells for 24 h. The cells were then lysed and EGFP proteins were pulled-down using GFP-Trap (ChromoTek). Proteins in the input lysate and in the pull-down output fractions were analyzed by immunoblotting using anti-HA and anti-GFP antibodies. **(A)** EGFP-CCDC146_692−955_ and EGFP-CCDC146_FL_, but not EGFP alone, co-immunoprecipitate CT288_Δ*NΔTMD*_-HA. **(B)** EGFP-CT288_Δ*NΔTMD*_, but not EGFP alone, co-immunoprecipitates CCDC146_FL_-HA and CCDC146_692−955_-HA. The arrows indicate the position in the blots of the relevant proteins (CT288_Δ*NΔTMD*_-HA, predicted molecular mass of 50 kDa; EGFP, 28 kDa; EGFP-CCDC146_692−955_, 60 kDa; EGFP-CCDC146_FL_, 140 kDa; EGFP-CT288_Δ*NΔTMD*_, 80 kDa; CCDC146_FL_-HA, 110 kDa; CCDC146_692−955_-HA, 30 kDa). The whole blots are shown in Figures S2, S3.

To further test the interaction between CT288 and CCDC146, we performed co-immunoprecipitation (co-IP) assays after transient expression of the proteins in HEK293T cells. For this, we used plasmids encoding CT288_Δ*NΔTMD*_, CCDC146_FL_, and CCDC146_692−955_ either fused to the C-terminus of EGFP (EGFP-CT288_Δ*NΔTMD*_, EGFP- CCDC146_FL_, and EGFP-CCDC146_692−955_) or with a HA epitope tag at their C-termini (CT288_Δ*NΔTMD*_-HA, CCDC146_FL_-HA, and CCDC146_692−955_-HA). For the co-IP assays, we transfected HEK293T cells with combinations of two plasmids (one encoding an HA-tagged protein and one encoding either a EGFP fusion protein or EGFP alone, as control). The transfected cells were lysed and EGFP or EGFP fusion proteins were immunoprecipitated. This revealed that CT288_Δ*NΔTMD*_-HA was pulled-down by the IP of EGFP-CCDC146_692−955_ or of EGFP-CCDC146_FL_, but not by the IP of EGFP alone (Figure 2A and Figure S2). Similarly, CCDC146_692−955_-HA or CCDC146_FL_-HA were pulled-down by the IP of EGFP-CT288_Δ*NΔTMD*_ but not by EGFP alone (Figure 2B and Figure S3). Therefore, CT288 and CCDC146 can also interact after their ectopic expression in HEK293T cells. Furthermore, in these conditions, we could detect an interaction between CT288_Δ*NΔTMD*_ and CCDC146_FL_.

### CT288 produced by *C. trachomatis* during infection interacts with ectopically expressed EGFP-CCDC146_692−955_

We next tested if CT288 and CCDC146 could interact during *C. trachomatis* infection of HeLa cells. For this, we first constructed a *C. trachomatis* L2/434 strain carrying a plasmid encoding full-length CT288 with a double HA epitope tag at its C-terminus (pCT288-2HA). In this strain, expression of *ct288-2HA* is directed by the promoter of the gene encoding *C. trachomatis* IncD (Figure S4A). Immunoblotting of whole cell extracts of HeLa 229 cells left uninfected or infected by the *C. trachomatis* L2/434 strain or by L2/434 harboring pCT288-2HA revealed that CT288_FL_-2HA was expressed and migrated on SDS-PAGE as expected from its predicted molecular mass of 63 kDa (Figure S4B). Furthermore, indirect immunofluorescence microscopy using antibodies against HA and *C. trachomatis* major outer membrane protein (MOMP), or Inc protein CT442, of HeLa 229 cells infected for 24 h by *C. trachomatis* L2/434 harboring pCT288-2HA confirmed the localization of CT288_FL_-2HA at the inclusion membrane (Figure S4C). The intracellular multiplication of *C. trachomatis* L2/434 harboring pCT288-2HA was slightly, but significantly, affected relative to *C. trachomatis* L2/434, as judged by quantification of the number of infectious progeny of either strain at different times of infection of HeLa cells (Figure S4D). By comparison to *C. trachomatis* L2/434, chlamydial intracellular multiplication was also similarly affected in L2/434 strains transformed with plasmids expressing CT288_FL_-2HA from the tetracycline-inducible *tetA* promoter or from the *ct288* promoter (data not shown).

**Figure 3:**
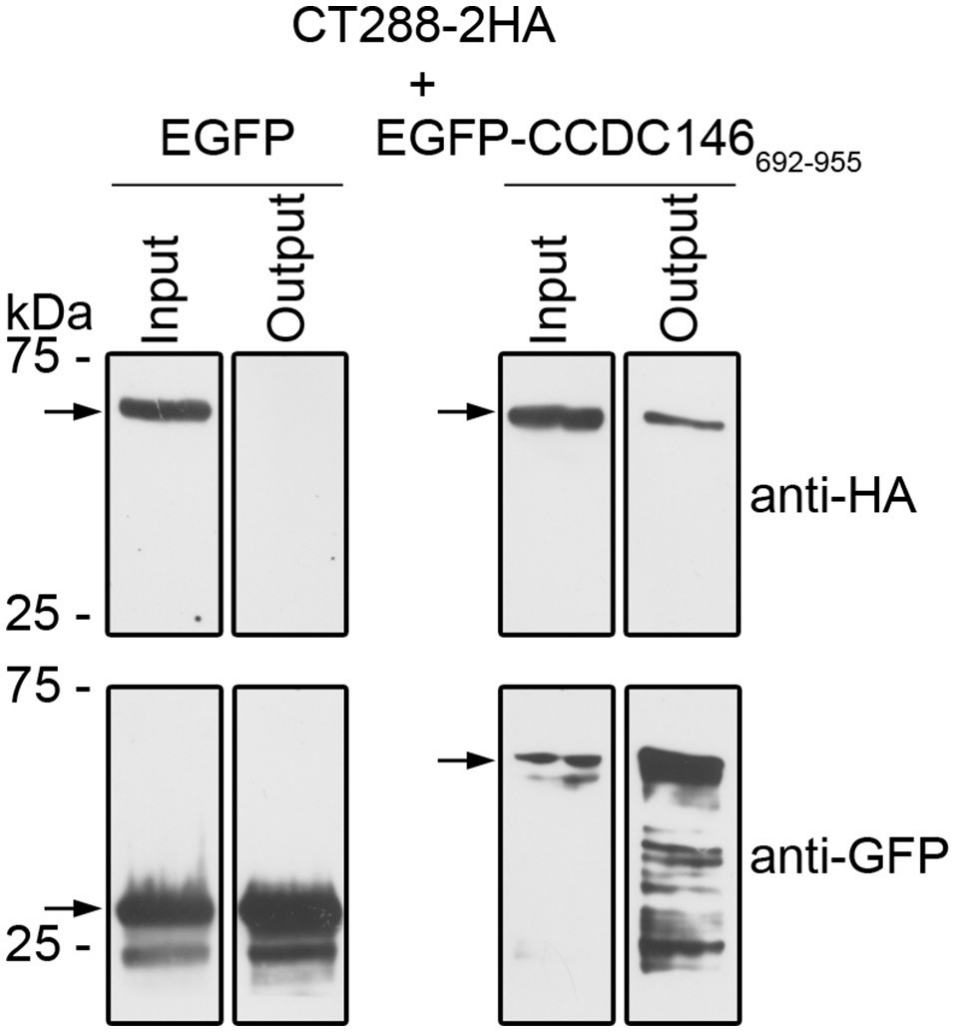
CT288-2HA produced by *C. trachomatis* during infection interacts with EGFP-CCDC146_692−955_. Plasmids encoding EGFP or EGFP-CCDC146_692−955_ were used to transfect HEK293T cells that were also infected for 24 h with *C. trachomatis* harboring pCT288-2HA. The cells were then lysed and EGFP proteins were pulled-down using GFP-Trap (ChromoTek). Proteins in the input lysate and in the pull-down output fractions were analyzed by immunoblotting using anti-HA and anti-GFP antibodies. The arrows indicate the position in the blots of the relevant proteins (CT288-2HA, predicted molecular mass of 66 kDa; EGFP, 28 kDa; EGFP-CCDC146_692−955_, 60 kDa). The whole blots are shown in Figure S5.

To test if CT288 and CCDC146 could interact during infection, we infected HEK293T cells with *C. trachomatis* L2/434 harboring pCT288-2HA. After the inoculation with *C. trachomatis*, HEK293T cells were also transfected with plasmids encoding EGFP or EGFP-CCDC146_692−955_. After 24 h of infection, the cells were incubated with 1% (w/v) paraformaldehyde as a crosslinking agent, lysed, and EGFP or EGFP-CCDC146_692−955_ were immunoprecipitated. Subsequent immunoblotting revealed that CT288_FL_-2HA was pulled-down by the IP of EGFP-CCDC146_692−955_ but not by EGFP (Figure 3 and Figure S5). Despite of multiple attempts, we were unable to detect an interaction between CT288_FL_-2HA and ectopically expressed EGFP-CCDC146_FL_ in *Chlamydia*-infected cells. Furthermore, as the endogenous levels of CCDC146 could not be detected in either HeLa or HEK293T cells by immunoblotting using a commercially available antibody that detected ectopically expressed EGFP-CCDC146_FL_ or CCDC146_FL_-HA (Figure S6), we could not test the interaction of endogenous CCDC146 with CT288.

Overall, these experiments support that *C. trachomatis* Inc CT288 can interact with the human centrosomal protein CCDC146.

### Ectopically expressed CCDC146 is recruited to the periphery of the inclusion membrane

**Figure 4:**
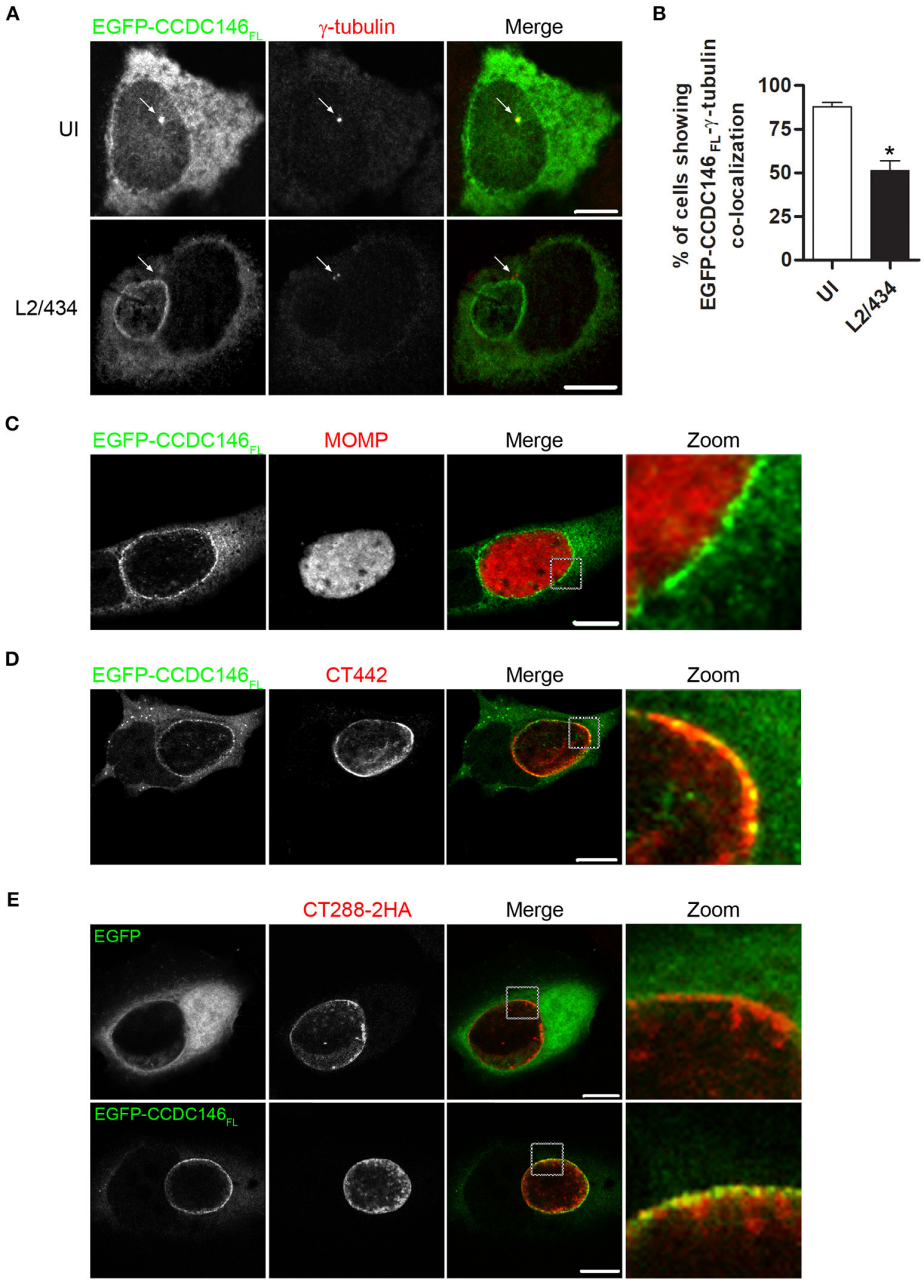
EGFP-CCDC146 is recruited to the periphery of the inclusion membrane in *C. trachomatis* infected cells. HeLa cells transfected with plasmids encoding either EGFP-CCDC146_FL_ or EGFP were left uninfected (UI) or infected for 24 h with *C. trachomatis* L2/434 **(A–D)** or *C. trachomatis* L2/434 harboring pCT288-2HA **(E)**. **(A)** The cells were fixed with methanol, immunolabeled with anti-GFP and anti-γ-tubulin antibodies, and appropriate fluorophore-conjugated secondary antibodies, and analyzed by confocal immunofluorescence microscopy. The arrows in each panel highlight the γ-tubulin-labeled centrosome. **(B)** Percentage of uninfected or *C. trachomatis*-infected HeLa 229 cells showing co-localization between EGFP-CCDC146_FL_ and γ-tubulin. Data represents three independent experiments (100 cells counted per experiment). *P*-values were calculated by a two-tailed unpaired Student's *t*-test relative to UI cells. ^*^P < 0.05. **(C–E)** The cells were fixed with paraformaldehyde 4% (w/v), immunolabeled with anti-MOMP **(C)**, anti-CT442 **(D)**, or anti-HA **(E)** antibodies, and appropriate fluorophore-conjugated secondary antibodies, and analyzed by confocal immunofluorescence microscopy. All scale bars, 10 μm.

To analyse the subcellular localization of ectopically expressed CCDC146, HeLa 229 cells were transiently transfected with plasmids encoding EGFP-CCDC146_FL_ or CCDC146_FL_-HA. Analysis of the transfected cells by indirect immunofluorescence microscopy with antibodies against GFP or HA and γ-tubulin (to label the centrosomes) revealed that in ~ 90% of the cells both EGFP-CCDC146_FL_ (Figures 4A,B) and CCDC146_FL_-HA (Figures S7A,B) could be co-localized with γ-tubulin, although the proteins were also seen dispersed in the cytosol. However, in cells transfected with plasmids encoding EGFP-CCDC146_FL_ or CCDC146_FL_-HA and infected for 24 h by *C. trachomatis* L2/434, the co-localization between γ-tubulin and EGFP-CCDC146_FL_ (Figures 4A,B) or CCDC146_FL_-HA (Figures S7A,B) was less evident and could only be seen in about 40% of the cases. Instead, in virtually all infected cells, ectopically expressed EGFP-CCDC146_FL_ or CCDC146_FL_-HA showed a ring-like appearance suggestive of their accumulation in the periphery of the inclusion (Figure 4A and Figure S7A). To further analyse this, cells transiently expressing EGFP-CCDC146_FL_ and infected by the L2/434 strain were analyzed by indirect confocal immunofluorescence microscopy using anti-MOMP antibodies or anti-Inc CT442 antibodies, which confirmed that EGFP-CCDC146_FL_ was around the inclusion (Figure 4C) and co-localized with CT442 (Figure 4D). Similar observations were made in cells expressing CCDC146_FL_-HA and infected by the L2/434 strain (Figures S7C,D). We also examined the localization of ectopically expressed EGFP-CCDC146_FL_, or EGFP alone as control, in cells infected by *C. trachomatis* L2/434 harboring pCT288-2HA. As expected from the previous analyses (Figures 4A–C), EGFP-CCDC146_FL_, but not EGFP alone, localized at the periphery of the inclusion membrane and showed colocalization with bacterially translocated CT288-2HA (Figure 4E).

In summary, these experiments showed that in *Chlamydia*-infected cells ectopically expressed CCDC146 is recruited to the periphery of the inclusion membrane.

### Recruitment of full-length CCDC146 to the periphery of the inclusion membrane is not dependent on CT288

**Figure 5:**
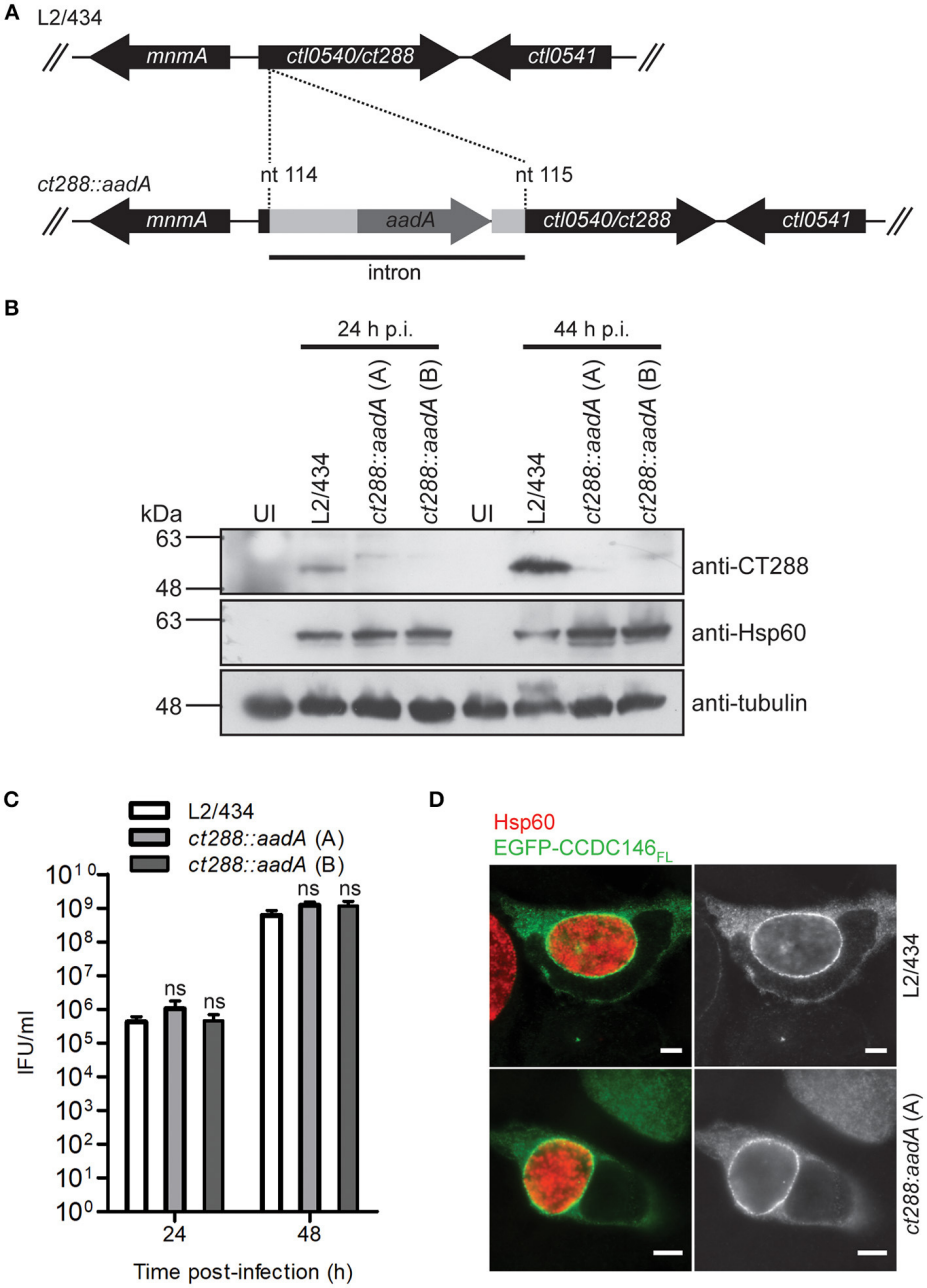
Recruitment of full-length CCDC146 to the periphery of the *C. trachomatis* inclusion does not depend on CT288. **(A)** A *C. trachomatis ctl0540* (*ct288* in *C. trachomatis* strain D/UW3) mutant was generated in strain L2/434 by the targeted insertion of a modified group II intron carrying the *aadA* gene, conferring spectinomycin resistance. **(B)** HeLa cells were infected for 24 or 44 h (p.i., post-infection) with *C. trachomatis* L2/434 or with two *C. trachomatis ct288:aadA* insertional mutant plaque-purified clones **(A,B)**. Whole cell lysates were analyzed by immunoblotting with antibodies against CT288, *C. trachomatis* Hsp60 (bacterial loading control) and α-tubulin (loading control for host cells). **(C)** HeLa cells were infected with the indicated strains at a multiplicity of infection of 5 and recoverable inclusion forming units (IFUs) were determined at 24 and 48 h p.i., Data are mean and standard error of the mean of 4 independent experiments. For each time-point, *P*-values were calculated by a two-tailed unpaired Student's *t*-test relative to the L2/434 strain; ns, not significant. **(D)** HeLa cells transfected with plasmids encoding EGFP or EGFP-CCDC146_FL_, and infected for 24 h by *C. trachomatis* L2/434 or *ct288:aadA* (clone A) were fixed with methanol, immunolabeled with anti-GFP and anti-Hsp60 antibodies, and appropriate fluorophore-conjugated secondary antibodies, and analyzed by immunofluorescence microscopy. Identical observations were made with clone B (data not shown). Scale bars, 5 μm.

To analyse if recruitment of CCDC146 to the periphery of the inclusion membrane is dependent on CT288, we constructed a *C. trachomatis* L2/434-derived strain where the *ct288* gene was inactivated by insertion between nucleotides 114 and 115 of a modified group II intron containing a spectinomycin-resistance gene (*aadA*) (Figure 5A). The correct insertion of the intron in the *ct288::aadA* mutant strain was verified by PCR and locus-specific DNA sequencing (Figure S8). This insertion could lead to the production of a truncated peptide of 49 amino acid residues, lacking the transmembrane regions of CT288 and with 11 of the residues encoded by the intron nucleotide sequence. To further characterize the insertional mutant strain, immunoblotting of extracts of HeLa cells infected by L2/434 or by *ct288::aadA* confirmed that insertion of the intron prevented detectable production of full-length CT288 (Figure 5B). A previous report where a similar penicillin-resistant *ct288::bla* mutant was constructed and analyzed, revealed a slight intracellular growth defect associated with the *ct288* mutant strain (Weber et al., 2017). However, the intracellular growth of *C. trachomatis ct288::aadA* was not significantly different from the parental L2/434 strain (Figure 5C). In HeLa cells transfected with the plasmid encoding EGFP-CCDC146_FL_ and infected for 24 h either by the L2/434 strain or by the *ct288::aadA* mutant strain, we observed recruitment of EGFP-CCDC146_FL_ to the periphery of the inclusion membrane in both cases (Figure 5D). EGFP-CCDC146_FL_ was first detected around the inclusion at 16 h p.i., in cells infected by the L2/434 strain (Figure S9A), and the same was observed in cells infected by the *ct288::aadA* mutant strain (Figure S9B). We also tested if presence of ectopically expressed EGFP-CCDC146_FL_ at the periphery of the inclusion would be affected by disrupting host cell microtubules (with nocodazole), microfilaments (with cytochalasin D), or the Golgi complex (with BFA), and whether this would depend on CT288. However, in *Chlamydia*-infected cells, regardless of the presence of intact *ct288*, EGFP-CCDC146_FL_ remained at the periphery of the inclusion after disruption of the cytoskeleton or Golgi (Figure S10A). Likewise, when adding the same drugs at 8 h p.i., and fixing the cells at 16 h p.i., we observed that EGFP-CCDC146_FL_ was recruited to the periphery of the inclusion in cells infected by the parental or mutant strain (Figure S10B). However, when chloramphenicol was added to block protein bacterial synthesis at 8 h p.i., then EGFP-CCDC146_FL_ was not detected around the inclusion at 16 h p.i., in cells infected by either of the strains (Figure S10C).

In summary, despite the ability of CT288 to interact with CCDC146 (Figures 1–3), recruitment of ectopically expressed CCDC146 to the periphery of the inclusion in *C. trachomatis*-infected cells does not depend on the presence of CT288 (Figures 4, 5). Recruitment of ectopically expressed EGFP-CCDC146_FL_ to the periphery of the inclusion does not require intact host Golgi, microtubules, or microfilaments, but depends on chlamydial protein synthesis.

### CT288 modulates the recruitment of ectopically expressed EGFP-CCDC146_692−955_ to the periphery of the inclusion

**Figure 6:**
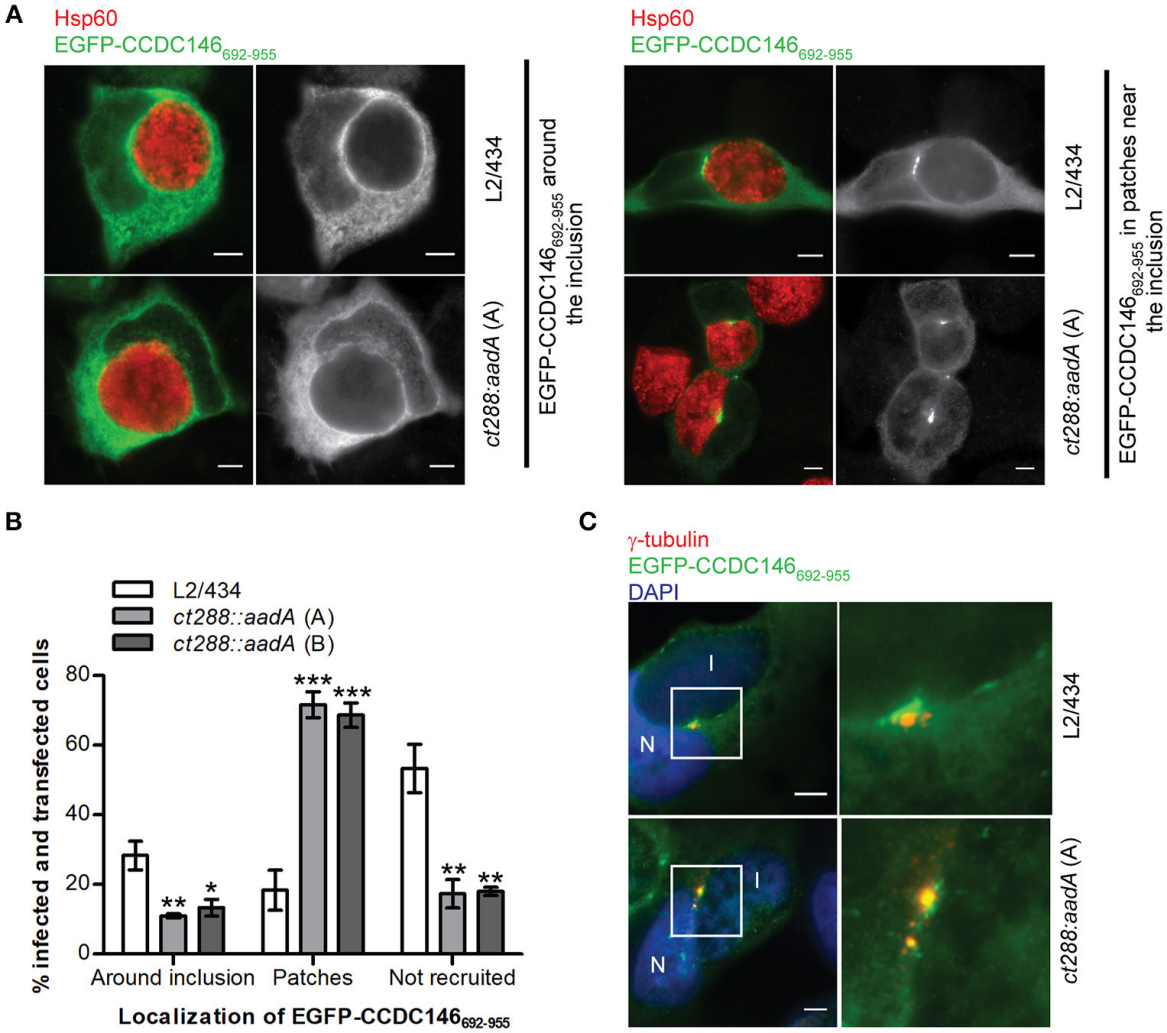
Recruitment of EGFP-CCDC146_652−955_ to the periphery of the *C. trachomatis* inclusion is modulated by CT288. HeLa cells transfected with a plasmid encoding EGFP- CCDC146_652−955_ were infected for 24 h with *C. trachomatis* L2/434 or the *ct288::aadA* mutant (clones A and B; see Figure 5). The cells were fixed with methanol, immunolabelled with anti-GFP, anti-γ-tubulin, or anti-Hsp60 antibodies, and appropriate fluorophore-conjugated secondary antibodies, and/or stained with DAPI (as indicated), and analyzed by immunofluorescence microscopy. **(A)** Representative images of the localization of ectopically expressed EGFP-CCDC146_652−955_ surrounding the inclusion (left-side panels), or in patches near the inclusion (right-side panels), in *Chlamydia*-infected cells. **(B)** Enumeration of cells infected by either *C. trachomatis* L2/434 or the *ct288::aadA* mutant showing ectopically expressed EGFP-CCDC146_652−955_ surrounding the inclusion, in patches near the inclusion, or not recruited to the inclusion periphery. Data represents three independent experiments (at least 50 infected and transfected cells were counted per experiment). *P*-values were obtained by one-way ANOVA and Dunnett *post-hoc* analyses relative to cells infected by the L2/434 strain in each class (around inclusion, in patches, or not recruited). ^*^P < 0.05; ^**^P < 0.01; ^***^P < 0.001. **(C)** Co-localization between EGFP-CCDC146_652−955_ in patches near the inclusion and γ-tubulin, in cells infected by *C. trachomatis* L2/434 (less frequently observed; see **B**) or the *ct288::aadA* mutant (more frequently observed; see **B)**. I, inclusion; N, host cell nucleus. Scale bars, 5 μm.

A fragment of CCDC146 between amino acids residues 692 and 955 was the minimal region of the protein that we have found to bind CT288 (Figures 1–3). We therefore tested if ectopically expressed EGFP-CCDC146_692−955_ was recruited to the periphery of the inclusion and if this was dependent on CT288. In *C. trachomatis*-infected cells, EGFP-CCDC146_692−955_ was more frequently found surrounding the inclusion of the L2/434 strain (28 ± 4%) than of the *ct288::aadA* mutant strain (11 ± 1%, clone A; 13 ± 2%, clone B) (Figures 6A,B). However, localization of ectopically expressed EGFP-CCDC146_692−955_ around the inclusion (Figure 6A) was much less evident than with EGFP-CCDC146_FL_ (Figures 4, 5D). No detectable recruitment of EGFP-CCDC146_692−955_ to the inclusion periphery was also more frequently seen in cells infected by the parental strain (53 ± 7%) than by the *ct288* mutant strain (17 ± 4%, clone A; 18 ± 1%, clone B) (Figures 6A,B). This is because in *C. trachomatis*-infected cells, EGFP-CCDC146_692−955_ was seen in patches near the inclusion (Figure 6A), which was much more frequent in cells infected by the *ct288::aadA* mutant (72 ± 4%, clone A; 69 ± 4%, clone B) than by the L2/434 strain (18 ± 6%; Figure 6B). This localization of EGFP-CCDC146_692−955_ in patches resembled the previously described inclusion microdomains that localize near host cell centrosomes (Mital et al., 2010). In fact, the patches of EGFP-CCDC146_692−955_ detected in cells infected by *C. trachomatis* were always very near of, or even co-localizing with, γ-tubulin-labeled centrosomes (Figure 6C).

In summary, these results indicate that: (i) EGFP-CCDC146_692−955_ can be recruited to the inclusion periphery partly, but not only, by CT288; (ii) the EGFP-CCDC146_692−955_-CT288 interaction is not sufficient to fully recruit EGFP-CCDC146_692−955_, either because EGFP-CCDC146_692−955_ is not always recruited or dissociates after recruitment; (iii) *Chlamydia* infection of HeLa cells promotes the accumulation of EGFP-CCDC146_692−955_ in patches near the inclusion but this is inhibited by CT288. Therefore, CT288 modulates the recruitment of EGFP-CCDC146_692−955_ to the periphery of the inclusion, which also suggests it could control the function of CCDC146 at the inclusion membrane during infection.

## Discussion

We found that the human centrosomal protein CCDC146 can bind the *C. trachomatis* Inc protein CT288 and is recruited to the periphery of the inclusion membrane in cells infected by *C. trachomatis*. This recruitment of CCDC146 is not CT288-dependent because it was observed both in cells infected by *C. trachomatis* strain L2/434 (producing CT288) and by a *C. trachomatis* mutant (*ct288::aadA*) that does not produce CT288. Furthermore, recruitment of CCDC146 did not require intact host Golgi, microtubules or microfilaments, but was dependent on chlamydial protein synthesis. This indicates that CCDC146 is recruited to the periphery of the inclusion membrane either directly or indirectly by other *Chlamydia* proteins. CCDC146 could bind directly other Inc proteins or interact with host cell proteins recruited by them to the inclusion membrane or to its periphery. 14-3-3β is another host protein recruited to the inclusion membrane that binds more than one Inc (at least, IncG and CT813/InaC) (Scidmore and Hackstadt, 2001; Kokes et al., 2015). Another example indicating the existence of multiple interactions, or redundant mechanisms, between Incs and host proteins is CT005/IncV, which binds and recruits VAPs (vesicle-associated membrane protein-associated proteins) to the inclusion membrane, but an *incV* mutant displays a moderate defect in VAP recruitment (Stanhope et al., 2017). In our case, we propose a model in which the described interaction between CT288 and CCDC146 would function to modulate the activity of CCDC146 at the inclusion membrane. This hypothesis is supported by the observations indicating that recruitment of ectopically expressed EGFP-CCDC146_692−955_ to the inclusion periphery is modulated by CT288. However, a role of CT288 in contributing to the recruitment of full-length CCDC146 to the inclusion membrane cannot be ruled out, as we analyzed ectopically expressed EGFP-CDCC146_FL_ and hypothetical CT288-dependent subtle effects might only be detectable by looking at the endogenous CCDC146.

CCDC146 has been shown to localize at the centrosome, in particular at the mother centriole (Firat-Karalar et al., 2014), and we could observe the centrosomal localization by ectopic expression of CCDC146 in HeLa cells and its co-localization with γ-tubulin. Although CCDC146 (NCBI Reference Sequence: NP_065930.2) possesses a predicted SMC (structural maintenance of chromosomes) region, the exact cellular roles of the protein are unknown. Unfortunately, it was not possible to address the physiological and functional relevance of the recruitment of CCDC146 to the periphery of the inclusion, because we could not detect production of the protein in the mammalian cell lines used (HeLa and HEK293T). This did not enable us to test if the CT288-CCDC146 interaction occurs under near physiological conditions, where both proteins are produced at endogenous levels, and prevented us from testing the consequences of depleting CCDC146 on the intracellular multiplication of *C. trachomatis*. The presumably very low levels of CCDC146 in HeLa and HEK293T cells also explain why the protein was not found in a previous quantitative proteomics analysis of the inclusion (Aeberhard et al., 2015), and why the CT288-CCDC146 interaction was not detected in the proteomics-based Inc-human interactome (Mirrashidi et al., 2015). However, a RNA sequencing (RNA-Seq) analysis of tissue samples from several human individuals revealed the highest levels of CCDC146 RNA in testis and above-average levels of CCDC146 RNA in the endometrium (Fagerberg et al., 2014), which suggests that the protein should be present in tissues related to *C. trachomatis* genital infections. Furthermore, CT288 is a core Inc protein (Lutter et al., 2012), with orthologues in different *Chlamydia* species, but not in environmental *Chlamydiae*, and CCDC146 is conserved among a large number of eukaryotes, including mammals, birds, and amphibians. Therefore, while this remains to be directly analyzed, it is possible that the CT288-CCDC146 interaction and recruitment of CCDC146 to the periphery of the inclusion are relevant in the general context of *Chlamydia* infections.

We constructed a *C. trachomatis ct288::aadA* mutant using the same parental strain, methodology and intron insertion site that had been previously used to generate a *ct288*::*bla* mutant (Weber et al., 2017). By comparison to the parental strain, we did not detect an intracellular growth defect for the *ct288::aadA* mutant. In contrast, a slight defect in intracellular growth has been reported for the *ct288::bla* mutant (Weber et al., 2017). Without a side-by-side comparison, the reason for the discrepancy in the analyses of the intracellular growth of the two *ct288* mutants are unclear, but they could be related to the different antibiotic resistance genes in the inserted intron, different media and serum composition, or other slightly different experimental procedures. Weber et al. also reported a defect of the *ct288::bla* during *in vivo* infection of mice (Weber et al., 2017), which we did not analyze for the *ct288::aadA* mutant. Nevertheless, either considering our observations or those from Weber et al, it appears that CT288 is largely dispensable for *C. trachomatis* intracellular growth in a tissue culture infection system. This could be thought as somewhat surprising knowing that *C. trachomatis* have undergone a process of genome reduction (Nunes and Gomes, 2014), which suggests that the large majority of its actual genes should have an important role during infection. However, other *C. trachomatis* mutants defective for Inc proteins or other chlamydial T3S effectors that have been characterized (e.g., *ct005*/*incV, ct179, ct224, ct695*/*tmeB, ct813/inaC, ct850*) also did not reveal an intracellular growth defect in tissue culture infection models (McKuen et al., 2017; Stanhope et al., 2017; Weber et al., 2017). This suggests that the essentiality of a *C. trachomatis* gene for infection could extend beyond a role in intracellular growth in tissue culture and that a more detailed phenotypic analysis of the *ct288* mutant is required to clarify the function(s) of the protein during infection.

We detected the interaction between CT288 and CCDC146 using different methods (Y2H and co-immunoprecipitations), different experimental systems (yeast, transfected cells, infected cells), and different tagged versions (e.g., GFP- or 2HA-tagged) of the full-length or truncated proteins. However, regarding CT288, we only used a full-length version (containing its transmembrane domains) produced by *C. trachomatis* in infected cells because we expected that in the other experimental systems the presence of these hydrophobic regions would direct the protein to membranes (or lead to protein aggregation) and prevent the detection of protein-protein interactions. For CCDC146, when we ectopically expressed the C-terminal region of the protein (CCDC146_692−955_), the CT288-CCDC146 interaction was detected by all methods and in all experimental systems. However, this does not necessarily mean that the CT288-CCDC146 interaction is only mediated by this C-terminal region of CCDC146, as we did not perform a detailed mapping of the regions (and predicted coiled-coil domains) of the protein that are involved in the interaction. The interaction between CT288 and CCDC146 using full-length CCDC146 could only be detected when both proteins were ectopically expressed in HEK293T cells. It is possible that the topology of the fusion proteins in the Y2H assay prevented the detection of this interaction. Regarding the co-immunoprecipitation assay from infected cells, the detection of the CT288-CCDC146_FL_ interaction was probably hampered by the low amounts of CT288-2HA that could be solubilized from the protein inserted in the inclusion membrane and the lower level of expression of EGFP-CCDC146_FL_ relative to EGFP-CCDC146_692−955_. It should also be noted that prolonged ectopic expression of CCDC146_FL_ or CCDC146_692−955_ was toxic to mammalian cells and for that reason we did not analyze the presence of the protein at the periphery of the inclusion for longer than 24 h of *C. trachomatis* infection.

In previous studies, plasmid-encoded and FLAG-tagged CT288 (expressed from the tetracycline promoter) delivered by *C. trachomatis* into the inclusion membrane has been shown to localize in inclusion microdomains (Weber et al., 2015). These microdomains are characterized by the presence of phosphorylated Src family kinases (seen as patches, by immunofluorescence microscopy) nearby host centrosomes (Mital et al., 2010). We consistently observed a homogenous distribution of both *Chlamydia*-delivered CT288-2HA and ectopically-expressed EGFP-CCDC146_FL_ around the inclusion, which did not suggest their localization at inclusion microdomains. However, we did detect ectopically expressed EGFP-CCDC146_692−955_ in patches near the inclusion and of host centrosomes labeled with γ-tubulin. This localization of EGFP-CCDC146_692−955_ was much more frequently seen in cells infected by the *ct288::aadA* mutant than by the parental L2/434 strain, indicating that CT288 interferes with the localization of the protein at the microdomains. Because recruitment of EGFP-CCDC146_FL_, and possibly also of EGFP-CCDC146_692−955_, to the inclusion periphery depends on other chlamydial proteins, this further suggests a complex and dynamic recruitment of CCDC146 to the inclusion that might involve its transient localization at microdomains in a process modulated by CT288.

The CT288-CCDC146 interaction can be integrated in a series of previous observations that establish an intricate relationship between *C. trachomatis* and its virulence proteins and the host cell centrosome, the main microtubule-organizing center in mammalian cells. It has been known that the *C. trachomatis* inclusion migrates toward the centrosome in a dynein-dependent, but dynactin-independent, manner and that this inclusion-centrosome association is maintained throughout infection (Grieshaber et al., 2003). Notably, infection of host cells by *C. trachomatis* leads to different types of centrosomal or mitotic spindle abnormalities (Grieshaber et al., 2006; Johnson et al., 2009; Knowlton et al., 2011), which have been suggested to be related with the known epidemiological link between *C. trachomatis* infection and cervical cancer (Knowlton et al., 2011). Furthermore, *C. trachomatis* host cell infection causes alterations in the microtubule network (Al-Zeer et al., 2014; Dumoux et al., 2015; Wesolowski et al., 2017). Some of the molecular players involved in these *C. trachomatis* inclusion-centrosome/microtubule interactions have been revealed: Inc proteins IncB, CT101, CT222, and CT850 associate with Src family kinases in inclusion microdomains that associate with centrosomes (Mital et al., 2010); the chlamydial protease CPAF is responsible for defects in the regulation of centrosome duplication and for inducing early mitotic exit (Brown et al., 2014); Inc CT850 binds dynein light chain, which likely promotes the positioning of the inclusion at the centrosome (Mital et al., 2015); and Inc CT223/IPAM binds the host centrosomal protein 170 kDa (CEP170) to subvert microtubule functions (Dumoux et al., 2015). In addition, in a large proteomics screen several Inc proteins have been shown to be capable of binding to host cell proteins with functions related to the centrosome (CT005, CT192, CT383) and with the eukaryotic cell division cycle (CT036, CT135, CT249, CT324, CT449, CT556, CT728, CT788). At the present, it is generally unclear how all these centrosome-related phenotypes and *C. trachomatis* proteins are related with each other to contribute to chlamydial infection. Therefore, it is also unknown how the CT288-CCDC146 interaction relates with all these previous observations. A more detailed future characterization of the *C. trachomatis ct288* mutant could provide insights regarding this issue.

In summary, we found a novel interaction between a *C. trachomatis* Inc protein (CT288) and a host cell protein (CCDC146), which is recruited to the periphery of the inclusion membrane and thus could play an important role during *Chlamydia* host cell infection. However, the relevance of our findings remains to be validated in more physiological infection models.

## Author contributions

FA performed the experiments, interpreted the data, wrote the article. MPL and ISP performed the experiments, interpreted the data. SVP performed the experiments. LJM conceived and designed the project, interpreted the data, wrote the article.

### Conflict of interest statement

The authors declare that the research was conducted in the absence of any commercial or financial relationships that could be construed as a potential conflict of interest.

## Supplementary material

Figure S1 CT288 from different *C. trachomatis* strains (L2/434, C/TW3, and E/Bour) also bind CCDC146 by Y2H. **(A)** The DNA encoding CT288 from *C. trachomatis* strains L2/434, C/TW3, and E/Bour was used to construct plasmids enabling production of myc-tagged fusions of CT288_Δ*NΔTMD*_ to the Gal 4 DNA-binding domain. The numbers above the blot indicate the predicted molecular mass of the corresponding fusion proteins. **(B)** Interaction between CT288_Δ*NΔTMD*_ from the indicated *C. trachomatis* strain and CCDC146_692−955_ by Y2H. Yeast growth as blue colonies (dark in the image) in high stringency QDO/X/A media indicates a protein-protein interaction. pGADT7 is the empty plasmid (used to generate the construct encoding CCDC146_692−955_) used as control in the Y2H assays.

Figure S2 Whole blots of the co-immunoprecipitation experiments to test if CT288_Δ*NΔTMD*_ is pulled-down by EGFP-CCDC146 fusion proteins after ectopic expression in mammalian cells. For details see Figure 2 legend. The arrows indicate the position in the blots of the relevant proteins. I, input fractions; O, output fractions.

Figure S3 Whole blots of the co-immunoprecipitation experiments to test if CCDC146-derived proteins are pulled-down by EGFP-CT288_Δ*NΔTMD*_ after ectopic expression in mammalian cells. For details see Figure 2 legend. The arrows indicate the position in the blots of the relevant proteins. I, input fractions; O, output fractions.

Figure S4 Characterization of a *C. trachomatis* strain harboring a plasmid encoding CT288-2HA expressed from the promoter of the inclusion membrane protein gene *incD*. **(A)** Schematic representation of the *ct288* gene present in the plasmid (pSVP255) introduced *C. trachomatis* L2/434 strain. P_*incD*_, *incD* promoter; T_*incD*_, *incD* terminator. **(B)** HeLa cells were either left uninfected (UI) or infected by the indicated *C. trachomatis* strains for 14, 24, or 40 h. Whole cell lysates were analyzed by immunoblotting with antibodies against HA, *C. trachomatis* MOMP (bacterial loading control) and α-tubulin (loading control for host cells). **(C)** Hela cells infected by *C. trachomatis* expressing CT288-2HA for 24 h were fixed with paraformaldehyde 4% (w/v), immunolabeled with anti-HA and anti-MOMP antibodies, or anti-Inc CT442 antibodies, and adequate fluorescent-conjugated secondary antibodies, and analyzed by immunofluorescence microscopy. Scale bar 10 μm. **(D)** HeLa cells were infected with the indicated strains at a multiplicity of infection of 5 and recoverable inclusion forming units (IFUs) were determined at 20, 24, 30, and 40 h p.i., Data are mean and standard error of the mean of 3 independent experiments. *P*-values were calculated by a two-tailed unpaired Student's *t*-test. ^*^*P* < 0.05; ns, not significant.

Figure S5 Whole blots of the co-immunoprecipitation experiments to test if C*. trachomatis*-produced CT288-2HA is pulled-down by EGFP- CCDC146_692−955._ For details see Figure 3 legend. The arrows indicate the position in the blots of the relevant proteins. I, input fractions; O, output fractions.

Figure S6 Endogenous CCDC146 cannot be detected in whole-cell extracts of HeLa or HEK293T cells. Plasmids encoding the indicated proteins were used to transfect HeLa 229 **(A)** or HEK293T cells **(B)** for 24 h. Whole cell extracts were collected, and proteins were analyzed by immunoblotting using anti-CCDC146, anti-HA, anti-GFP, and anti-α-tubulin antibodies.

Figure S7 CCDC146-HA is recruited to the periphery of the inclusion membrane in *C. trachomatis* infected cells. HeLa cells transfected with a plasmid encoding CCDC146_FL_-HA were either left uninfected (UI) or infected for 24 h with *C. trachomatis* L2/434. **(A)** The cells were fixed with methanol, immunolabeled with anti-HA and anti-γ-tubulin antibodies, and appropriate fluorophore-conjugated secondary antibodies, and analyzed by confocal immunofluorescence microscopy. The arrows in each panel highlight the γ-tubulin-labeled centrosome. **(B)** Percentage of uninfected or *C. trachomatis*-infected HeLa 229 cells showing co-localization between CCDC146_FL_-HA and γ-tubulin. Data is from one experiment (100 cells counted). **(C)** and **(D)** The infected cells were fixed with paraformaldehyde 4% (w/v), immunolabeled with anti-HA and anti-MOMP **(C)**, or anti-CT442 **(D)** antibodies, and analyzed by confocal immunofluorescence microscopy. All scale bars, 10 μm.

Figure S8 Verification of intron insertion in the *C. trachomatis ct288:aadA* mutant strain. **(A)** Representation of the *ctl0540* (ortolog of *ct288* in strain D/UW3) locus in *C. trachomatis* LGV serovar L2 strain 434/Bu (L2/434). **(B)** Representation of the *ctl0540* locus in the *ct288:aadA* mutant derivative of *C. trachomatis* L2/434. In **(A)** and **(B)** the arrows and numbers indicate the approximate hybridization position of DNA primers (Table S2) used in PCR reactions, yielding DNA products of the indicated length in base pairs (bp). **(C)** Agarose gel displaying the result from the PCR with the indicated primers (Table S2) and DNA templates; pML2 is the plasmid containing the intron targeting *ct288* (Table S1), used to generate the *ct288:aadA* strain; bp, base pairs.

Figure S9 Comparison of the localization of ectopically expressed full-length EGFP-CCDC146 in cells infected by *C. trachomatis* L2/434 or *ct288::aadA* mutant strains. HeLa cells transfected with a plasmid encoding full-length EGFP-CCDC146 (EGFP-CCDC146_FL_) were infected for 8, 16, or 24 h by *C. trachomatis* L2/434 **(A)** or *ct288:aadA* (clone **A**; Figure 5) **(B)**. The cells were fixed with methanol, immunolabeled with anti-GFP and anti-Hsp60 antibodies, and appropriate fluorophore-conjugated secondary antibodies, and analyzed by immunofluorescence microscopy. Scale bars, 5 μm.

Figure S10 Localization of full-length EGFP-CCDC146 at the periphery of the *C. trachomatis* inclusion does not require intact host Golgi, microtubules or microfilaments, but depends on chlamydial protein synthesis. HeLa cells transfected with a plasmid encoding full-length EGFP-CCDC146 (EGFP-CCDC146_FL_) were infected for 24 h **(A)** or 16 h **(B,C)** by *C. trachomatis* L2/434 or *ct288:aadA* (clone A; Figure 5). The cells were fixed with methanol, immunolabeled with anti-GFP and anti-Hsp60 antibodies, and appropriate fluorophore-conjugated secondary antibodies, and analyzed by immunofluorescence microscopy. At 23 h p.i. **(A)** or 8 h p.i., **(B)**, the cells were incubated in the presence of 1 μg/ml nocodazole (to depolymerize microtubules), 2 μM cytochalasin D (to depolymerize microfilaments), or 1 μg/ml brefeldin A (BFA; to disrupt the Golgi complex). **(C)** At 8 h p.i., the cells were incubated in the presence of 100 μg/ml chloramphenicol (to inhibit bacterial protein synthesis). The solvents (dimethyl sulfoxide or ethanol) did not affect the localization of EGFP-CCDC146_FL_ at the inclusion periphery, and the disrupting effect of nocodazole, cytochalasin D, and BFA was confirmed by fluorescence microscopy (not shown). Scale bars, 5 μm.

Table S1 Plasmids used in this work.

Table S2 DNA primers used in this work.

Table S3 Candidate binding partners of C. trachomatis Inc protein CT288 identified in a yeast two-hybrid screen using a HeLa cDNA library as bait.
