# Supplementary figures and images for: The Human Centrosomal Protein CCDC146 Binds Chlamydia trachomatis Inclusion Membrane Protein CT288 and Is Recruited to the Periphery of the Chlamydia-Containing Vacuole
Source: Front Cell Infect Microbiol. 2018 Jul 26;8:254. doi: 10.3389/fcimb.2018.00254 (PMC6070772; doi:10.3389/fcimb.2018.00254)

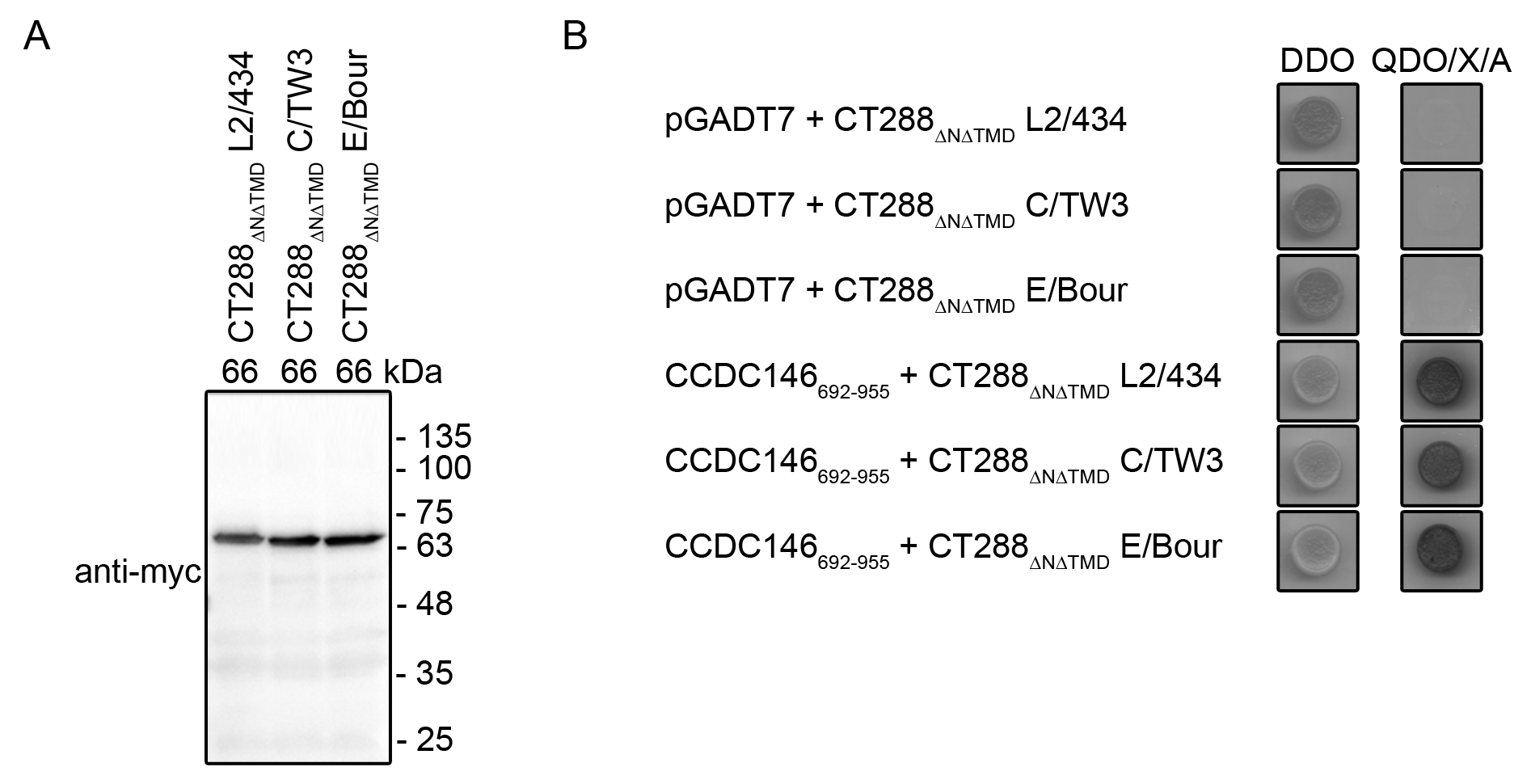

Supplement: Figure S1 — CT288 from different C. trachomatis strains (L2/434, C/TW3, and E/Bour) also bind CCDC146 by Y2H. (A) The DNA encoding CT288 from C. trachomatis strains L2/434, C/TW3, and E/Bour was used to construct plasmids enabling production of myc-tagged fusions of CT288ΔNΔTMD to the Gal 4 DNA-binding domain. The numbers above the blot indicate the predicted molecular mass of the corresponding fusion proteins. (B) Interaction between CT288ΔNΔTMD from the indicated C. trachomatis strain and CCDC146692−955 by Y2H. Yeast growth as blue colonies (dark in the image) in high stringency QDO/X/A media indicates a protein-protein interaction. pGADT7 is the empty plasmid (used to generate the construct encoding CCDC146692−955) used as control in the Y2H assays. [file Image_1.TIF]

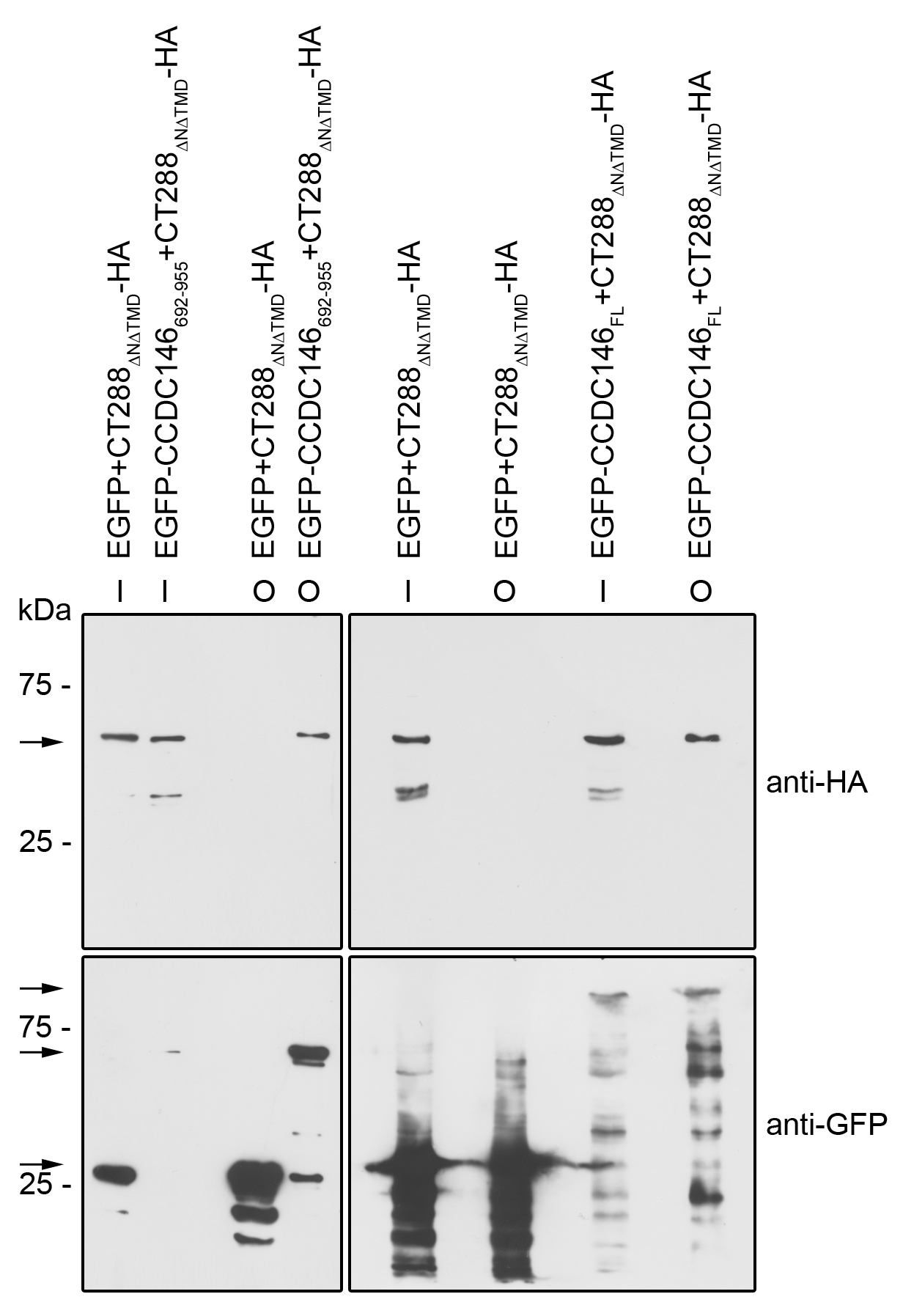

Supplement: Figure S2 — Whole blots of the co-immunoprecipitation experiments to test if CT288ΔNΔTMD is pulled-down by EGFP-CCDC146 fusion proteins after ectopic expression in mammalian cells. For details see Figure 2 legend. The arrows indicate the position in the blots of the relevant proteins. I, input fractions; O, output fractions. [file Image_2.TIF]

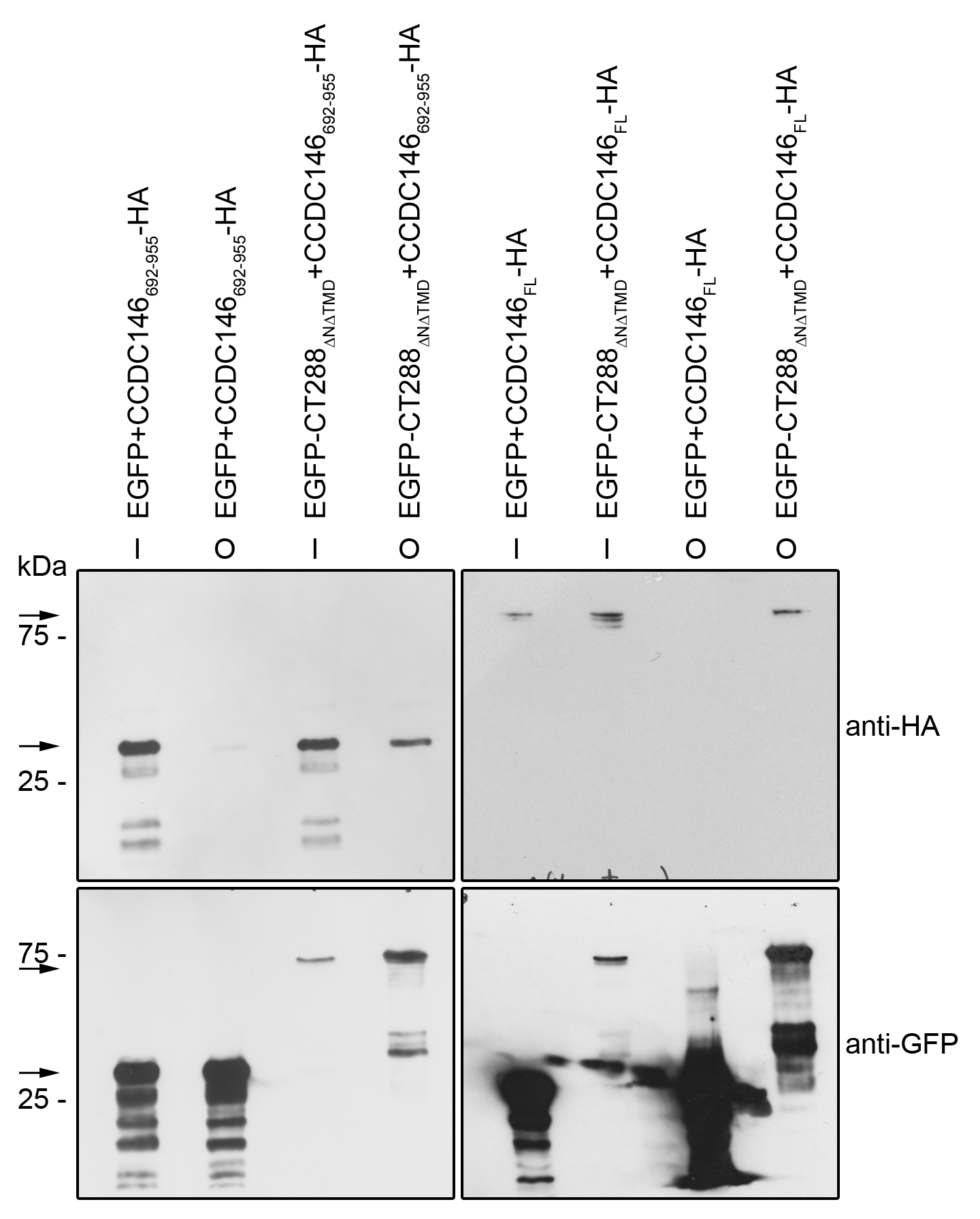

Supplement: Figure S3 — Whole blots of the co-immunoprecipitation experiments to test if CCDC146-derived proteins are pulled-down by EGFP-CT288ΔNΔTMD after ectopic expression in mammalian cells. For details see Figure 2 legend. The arrows indicate the position in the blots of the relevant proteins. I, input fractions; O, output fractions. [file Image_3.TIF]

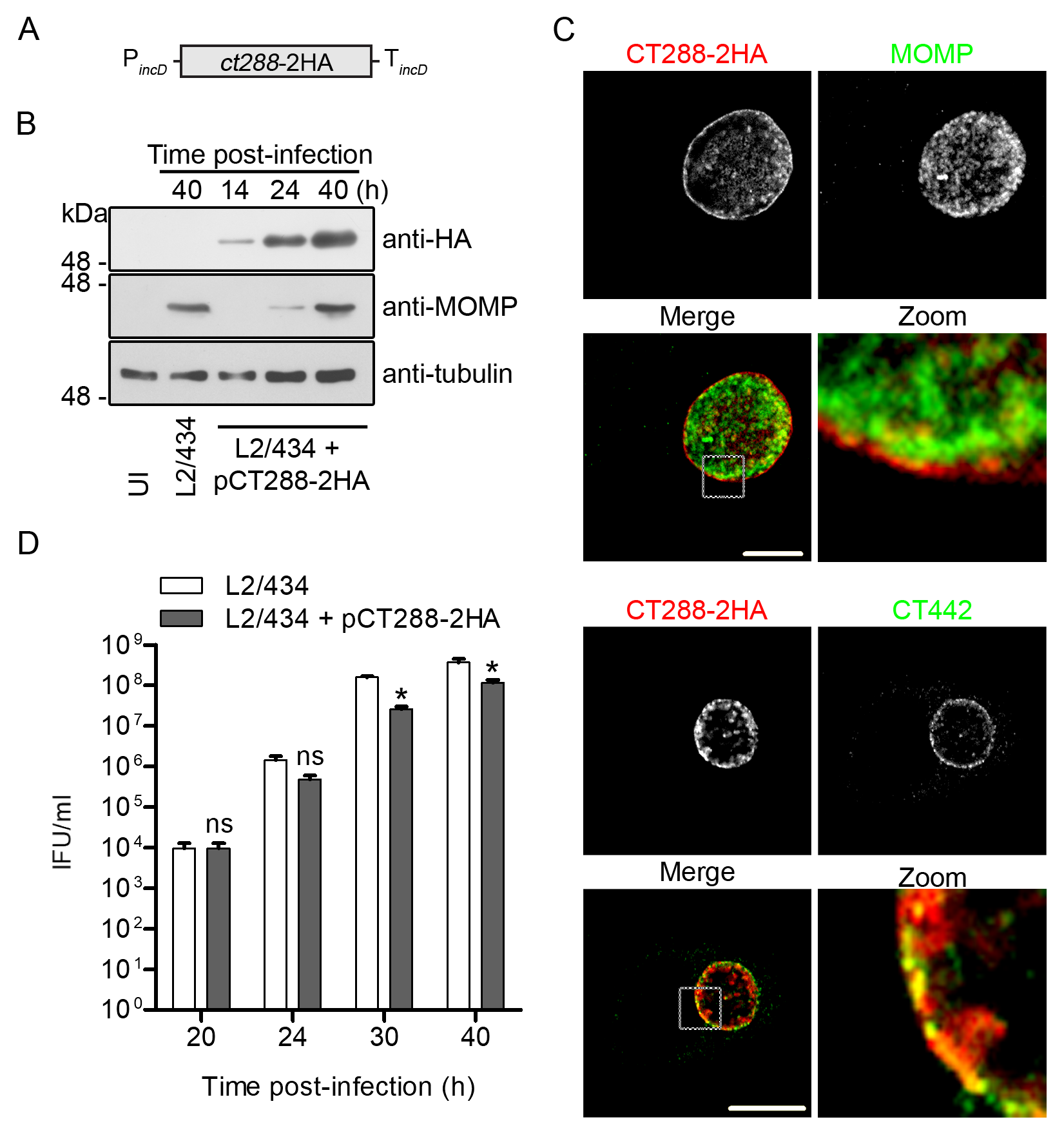

Supplement: Figure S4 — Characterization of a C. trachomatis strain harboring a plasmid encoding CT288-2HA expressed from the promoter of the inclusion membrane protein gene incD. (A) Schematic representation of the ct288 gene present in the plasmid (pSVP255) introduced C. trachomatis L2/434 strain. PincD, incD promoter; TincD, incD terminator. (B) HeLa cells were either left uninfected (UI) or infected by the indicated C. trachomatis strains for 14, 24, or 40 h. Whole cell lysates were analyzed by immunoblotting with antibodies against HA, C. trachomatis MOMP (bacterial loading control) and α-tubulin (loading control for host cells). (C) Hela cells infected by C. trachomatis expressing CT288-2HA for 24 h were fixed with paraformaldehyde 4% (w/v), immunolabeled with anti-HA and anti-MOMP antibodies, or anti-Inc CT442 antibodies, and adequate fluorescent-conjugated secondary antibodies, and analyzed by immunofluorescence microscopy. Scale bar 10 μm. (D) HeLa cells were infected with the indicated strains at a multiplicity of infection of 5 and recoverable inclusion forming units (IFUs) were determined at 20, 24, 30, and 40 h p.i., Data are mean and standard error of the mean of 3 independent experiments. P-values were calculated by a two-tailed unpaired Student's t-test. *P < 0.05; ns, not significant. [file Image_4.TIF]

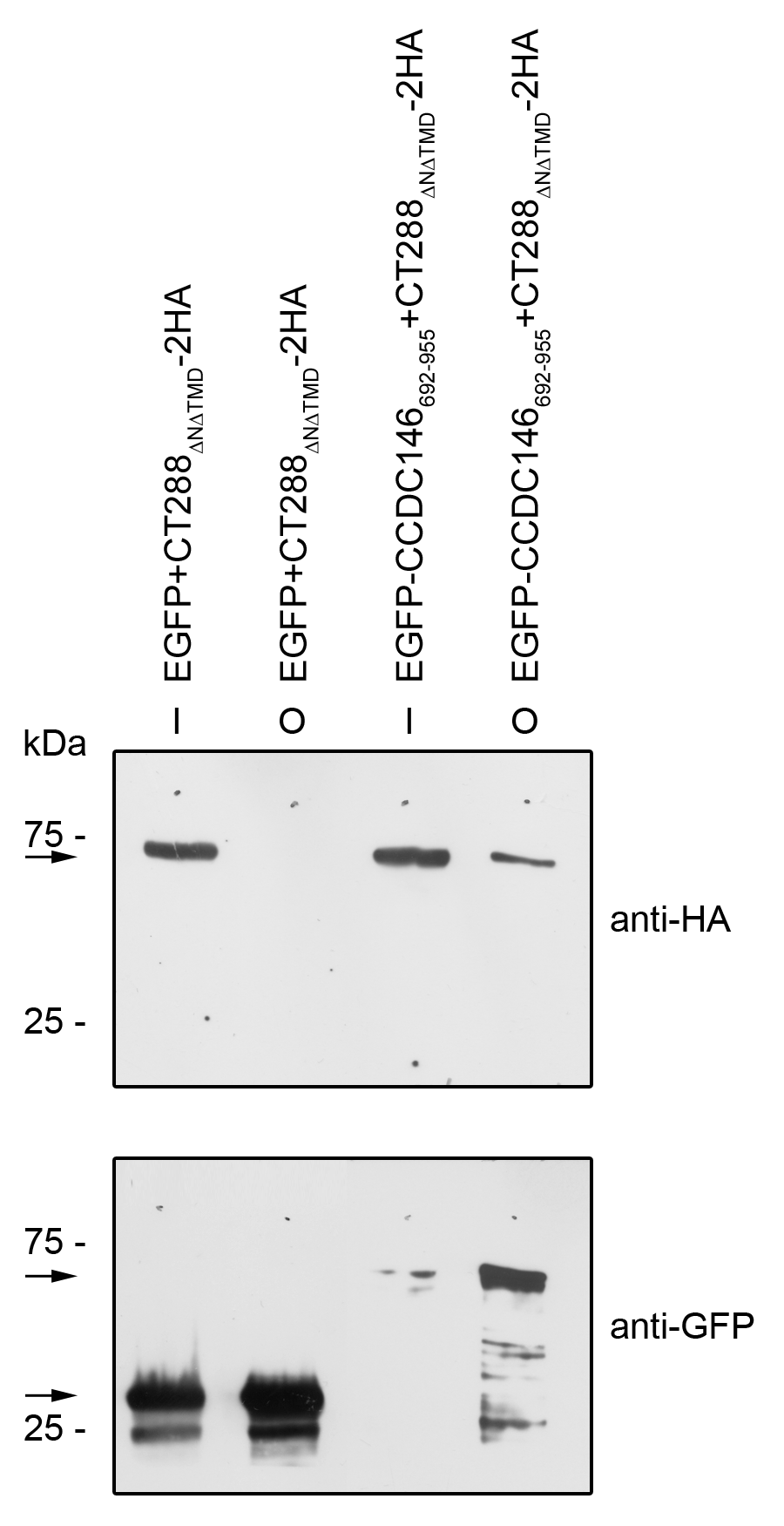

Supplement: Figure S5 — Whole blots of the co-immunoprecipitation experiments to test if C. trachomatis-produced CT288-2HA is pulled-down by EGFP- CCDC146692−955. For details see Figure 3 legend. The arrows indicate the position in the blots of the relevant proteins. I, input fractions; O, output fractions. [file Image_5.TIF]

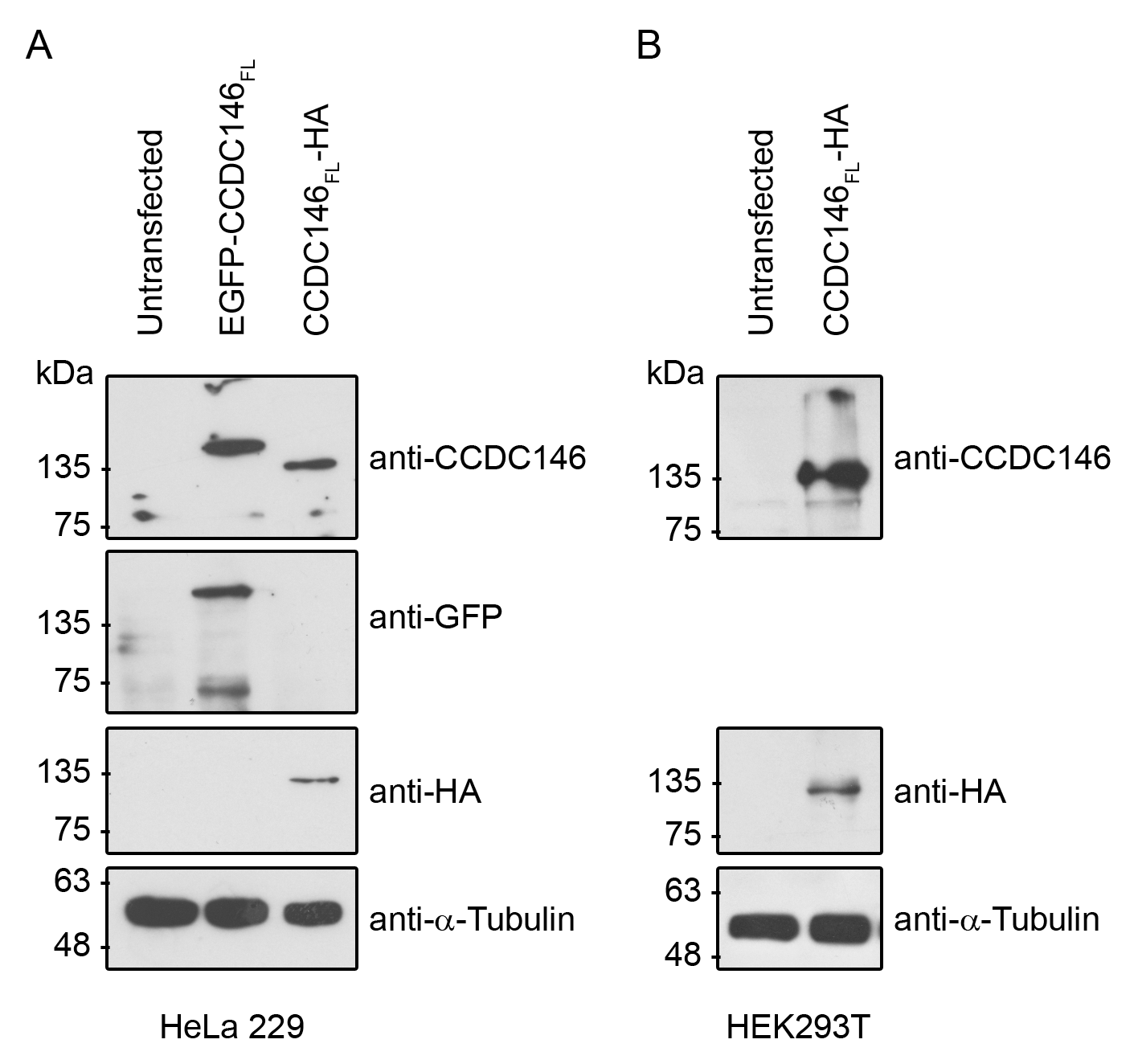

Supplement: Figure S6 — Endogenous CCDC146 cannot be detected in whole-cell extracts of HeLa or HEK293T cells. Plasmids encoding the indicated proteins were used to transfect HeLa 229 (A) or HEK293T cells (B) for 24 h. Whole cell extracts were collected, and proteins were analyzed by immunoblotting using anti-CCDC146, anti-HA, anti-GFP, and anti-α-tubulin antibodies. [file Image_6.TIF]

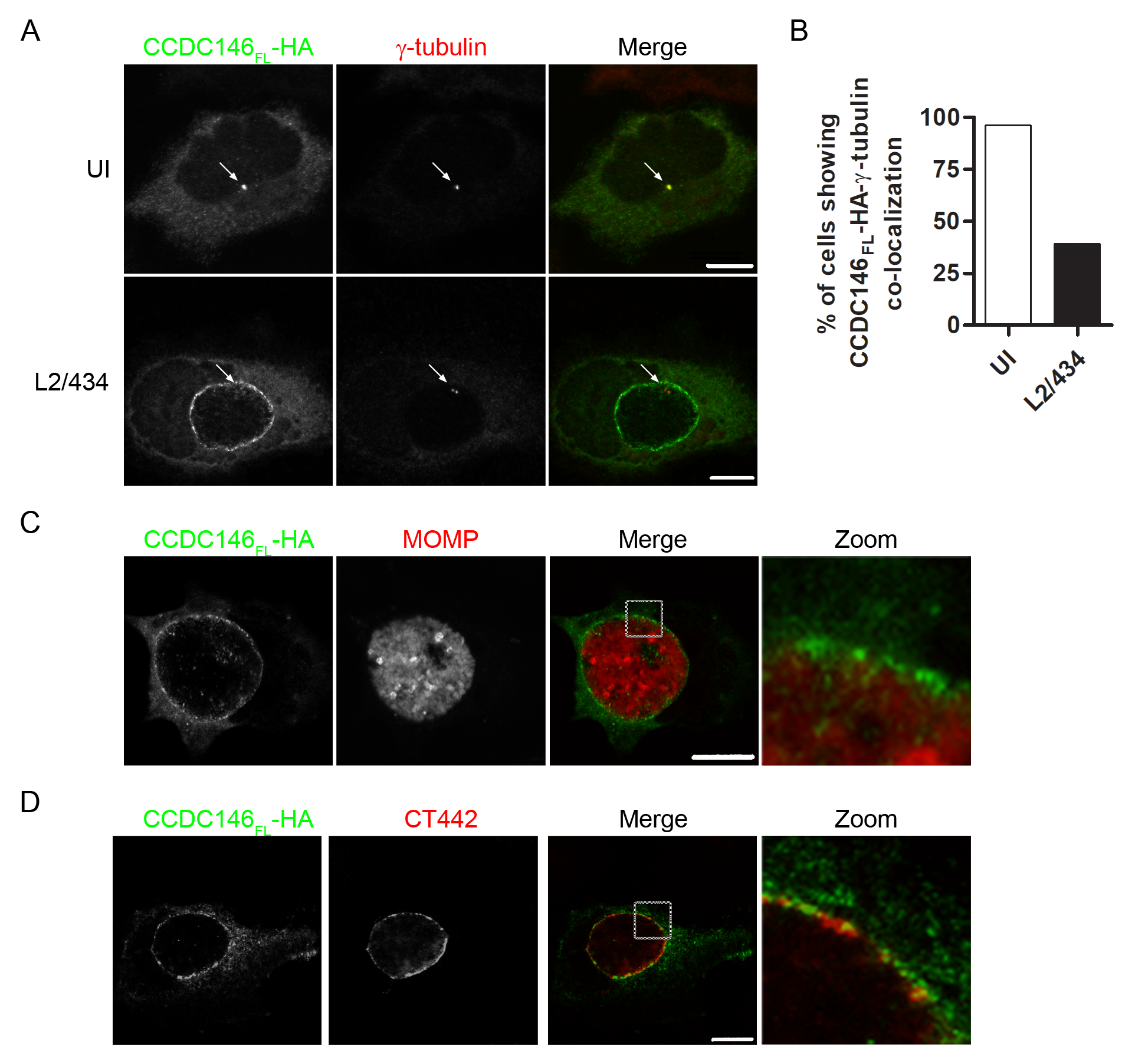

Supplement: Figure S7 — CCDC146-HA is recruited to the periphery of the inclusion membrane in C. trachomatis infected cells. HeLa cells transfected with a plasmid encoding CCDC146FL-HA were either left uninfected (UI) or infected for 24 h with C. trachomatis L2/434. (A) The cells were fixed with methanol, immunolabeled with anti-HA and anti-γ-tubulin antibodies, and appropriate fluorophore-conjugated secondary antibodies, and analyzed by confocal immunofluorescence microscopy. The arrows in each panel highlight the γ-tubulin-labeled centrosome. (B) Percentage of uninfected or C. trachomatis-infected HeLa 229 cells showing co-localization between CCDC146FL-HA and γ-tubulin. Data is from one experiment (100 cells counted). (C) and (D) The infected cells were fixed with paraformaldehyde 4% (w/v), immunolabeled with anti-HA and anti-MOMP (C), or anti-CT442 (D) antibodies, and analyzed by confocal immunofluorescence microscopy. All scale bars, 10 μm. [file Image_7.TIF]

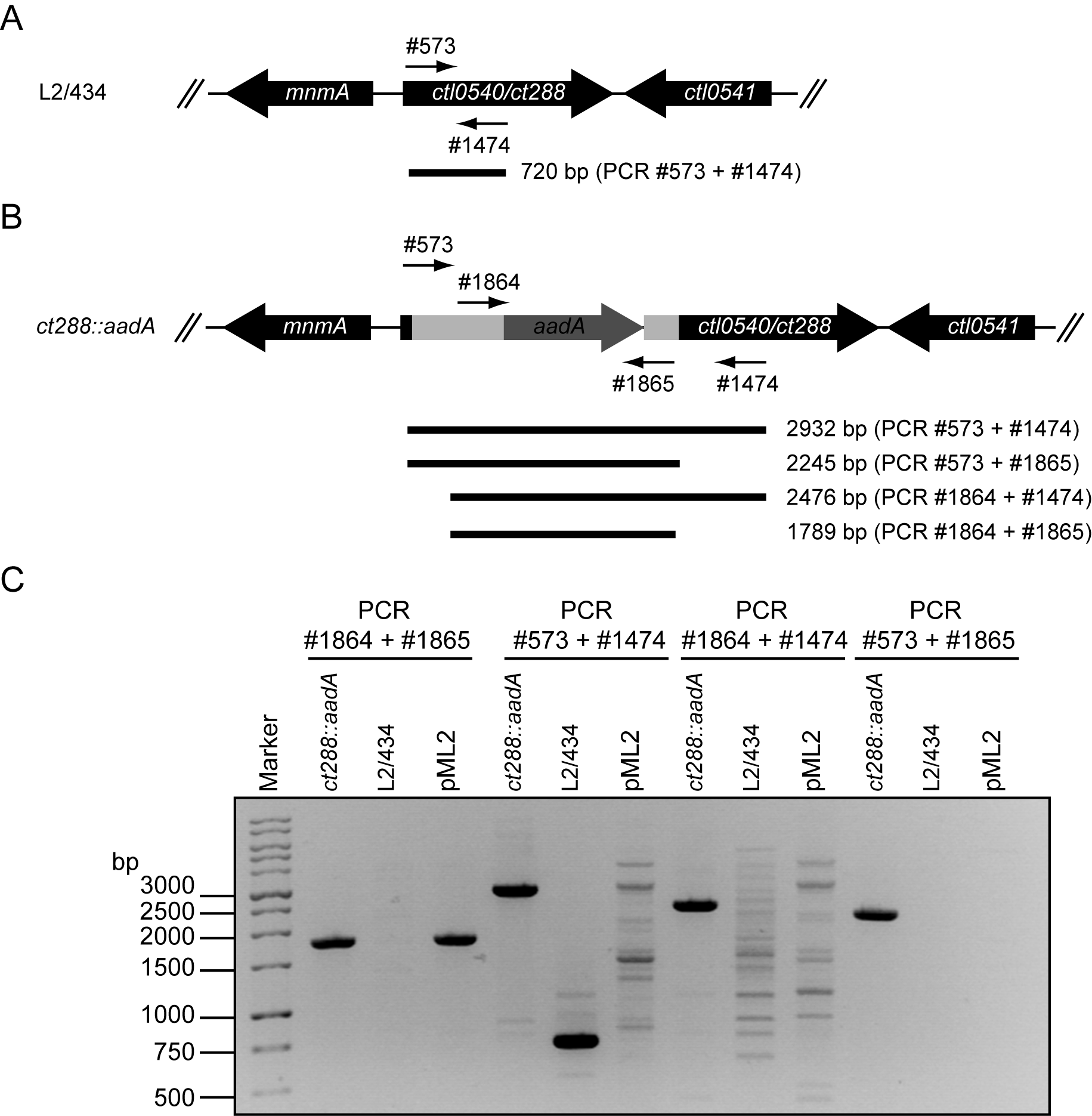

Supplement: Figure S8 — Verification of intron insertion in the C. trachomatis ct288:aadA mutant strain. (A) Representation of the ctl0540 (ortolog of ct288 in strain D/UW3) locus in C. trachomatis LGV serovar L2 strain 434/Bu (L2/434). (B) Representation of the ctl0540 locus in the ct288:aadA mutant derivative of C. trachomatis L2/434. In (A) and (B) the arrows and numbers indicate the approximate hybridization position of DNA primers (Table S2) used in PCR reactions, yielding DNA products of the indicated length in base pairs (bp). (C) Agarose gel displaying the result from the PCR with the indicated primers (Table S2) and DNA templates; pML2 is the plasmid containing the intron targeting ct288 (Table S1), used to generate the ct288:aadA strain; bp, base pairs. [file Image_8.TIF]

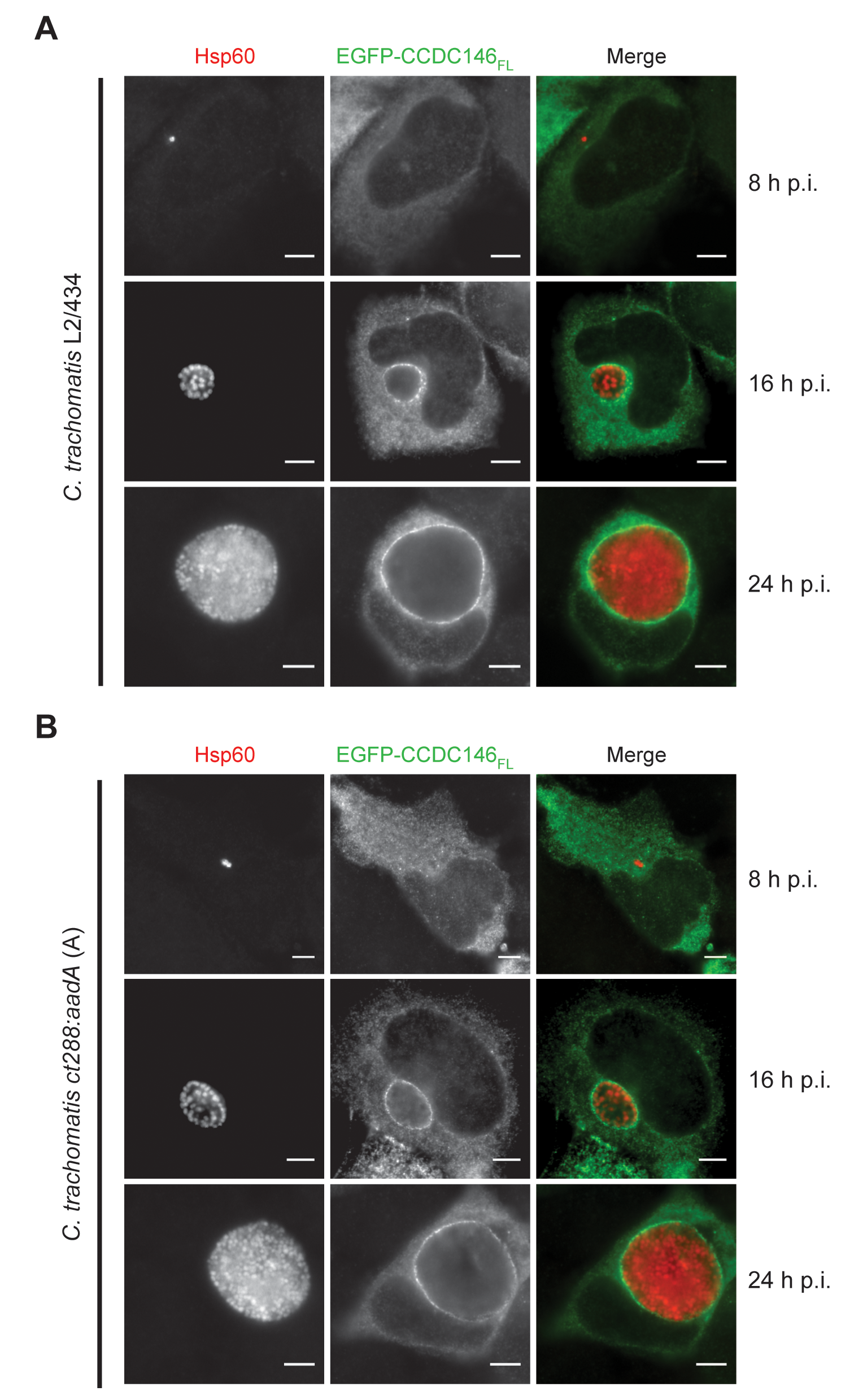

Supplement: Figure S9 — Comparison of the localization of ectopically expressed full-length EGFP-CCDC146 in cells infected by C. trachomatis L2/434 or ct288::aadA mutant strains. HeLa cells transfected with a plasmid encoding full-length EGFP-CCDC146 (EGFP-CCDC146FL) were infected for 8, 16, or 24 h by C. trachomatis L2/434 (A) or ct288:aadA (clone A; Figure 5) (B). The cells were fixed with methanol, immunolabeled with anti-GFP and anti-Hsp60 antibodies, and appropriate fluorophore-conjugated secondary antibodies, and analyzed by immunofluorescence microscopy. Scale bars, 5 μm. [file Image_9.TIF]

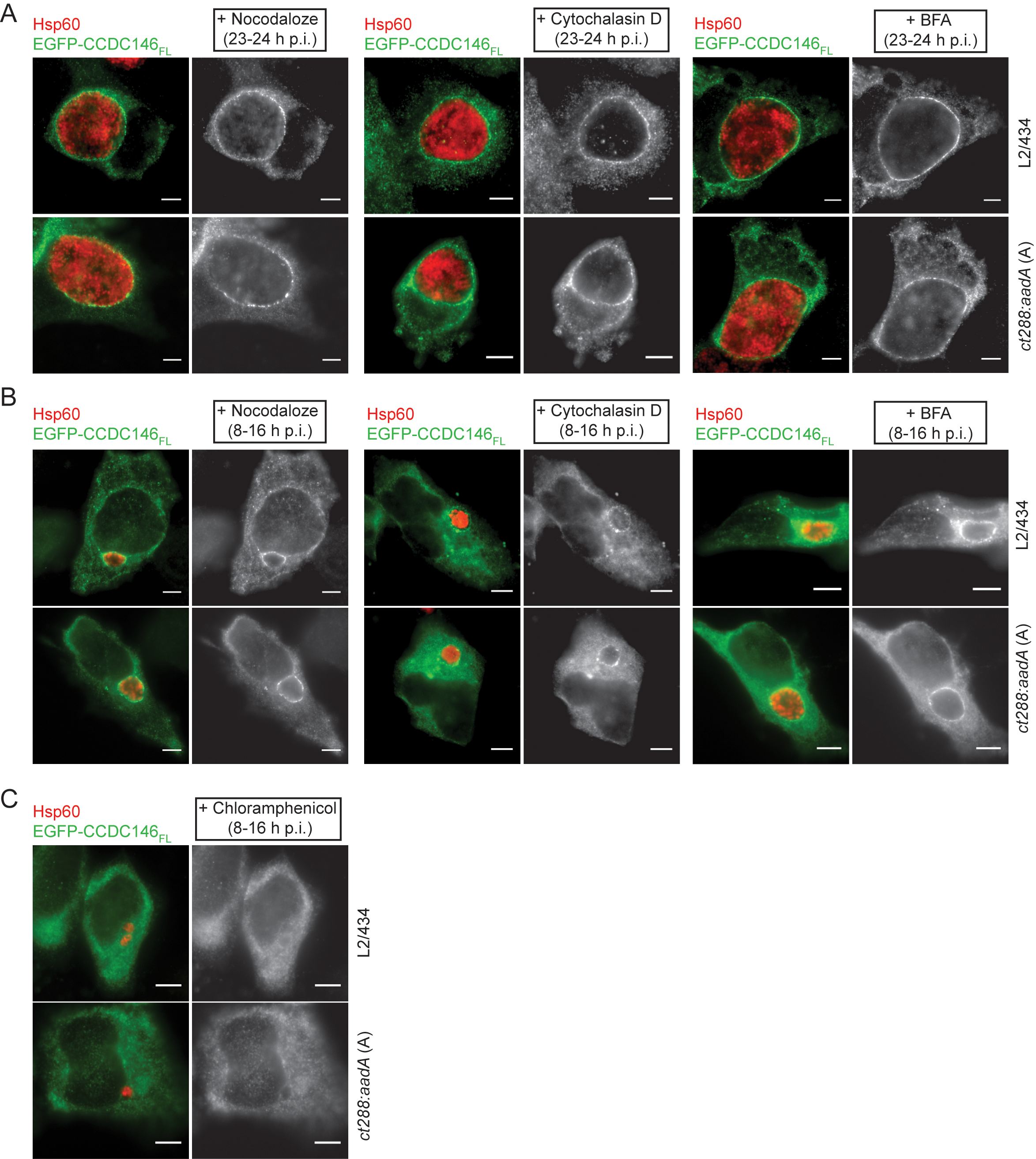

Supplement: Figure S10 — Localization of full-length EGFP-CCDC146 at the periphery of the C. trachomatis inclusion does not require intact host Golgi, microtubules or microfilaments, but depends on chlamydial protein synthesis. HeLa cells transfected with a plasmid encoding full-length EGFP-CCDC146 (EGFP-CCDC146FL) were infected for 24 h (A) or 16 h (B,C) by C. trachomatis L2/434 or ct288:aadA (clone A; Figure 5). The cells were fixed with methanol, immunolabeled with anti-GFP and anti-Hsp60 antibodies, and appropriate fluorophore-conjugated secondary antibodies, and analyzed by immunofluorescence microscopy. At 23 h p.i. (A) or 8 h p.i., (B), the cells were incubated in the presence of 1 μg/ml nocodazole (to depolymerize microtubules), 2 μM cytochalasin D (to depolymerize microfilaments), or 1 μg/ml brefeldin A (BFA; to disrupt the Golgi complex). (C) At 8 h p.i., the cells were incubated in the presence of 100 μg/ml chloramphenicol (to inhibit bacterial protein synthesis). The solvents (dimethyl sulfoxide or ethanol) did not affect the localization of EGFP-CCDC146FL at the inclusion periphery, and the disrupting effect of nocodazole, cytochalasin D, and BFA was confirmed by fluorescence microscopy (not shown). Scale bars, 5 μm. [file Image_10.TIF]
