# Supplementary material for: The Human Centrosomal Protein CCDC146 Binds Chlamydia trachomatis Inclusion Membrane Protein CT288 and Is Recruited to the Periphery of the Chlamydia-Containing Vacuole
Source: Front Cell Infect Microbiol. 2018 Jul 26;8:254. doi: 10.3389/fcimb.2018.00254 (PMC6070772; doi:10.3389/fcimb.2018.00254)
Supplement: Table S1 — Plasmids used in this work. [file Table_1.PDF]

**Table S1. Plasmids used in this work.**

| Plasmid | Description/Construction | Reference |
|---|---|---|
| pGADT7 | Yeast two-hybrid (Y2H) vector for expression of Gal4 activation domain (AD) and *myc* epitope tag fusion proteins (Gal4AD-*myc*). | Clontech |
| pGBKT7 | Y2H vector for expression of Gal4 DNA binding domain (BD) and HA epitope tag fusion proteins (Gal4BD-HA). | Clontech |
| pEGFP-C1 | Mammalian transfection vector for expression of EGFP fusion proteins under the control of the *CMV* promoter. | Clontech |
| pEF6/*myc*-His C | Mammalian transfection vector for expression of proteins under the control of the *EF-1α* promoter. | Thermo Fisher Scientific |
| pSVP247 | Modified *C. trachomatis*-*E. coli* shuttle vector for expression of double HA epitope (2HA)-tagged proteins, with transcription halted by the terminator of the gene encoding inclusion membrane protein D (*incD*). | Cunha et al, 2017 |
| pCCDC146 | Contains the cDNA of *CCDC146*; clone IRATp970G0632D. | Source BioScience |
| pDFTT3 aad | Group II intron donor plasmid. | Key and Fisher, 2017 |

**Table S1.** ***Continued.***

| Plasmid | Description/Construction | Reference |
|---|---|---|
| pSVP255/pCT288-2HA | Derivative of pSVP247 encoding full-length CT288 with a C-terminal 2HA tag ($CT288_{FL}$-2HA) under the control of the promoter of *incD* ($P_{incD}$). The *ct288* gene and the $P_{incD}$ were amplified by PCR from L2/434 chromosomal DNA using primers #1565 and #1567, and #1546 and #1566, respectively. The DNA products were then fused by overlapping PCR using primers #1546 and #1567. The final DNA product was digested with KpnI-NotI and ligated into those sites of pSVP247. | This work. |
| pCT288Int18 | pGADT7 derivative plasmid encoding a fusion of the Gal4AD-HA to the C-terminal region (amino acid residues 692 to 955) of CCDC146 (Gal4AD-HA-$CCDC146_{692\text{-}955}$) that was recovered from the Y2H screen using pFA147 as bait. | This work. |
| pFA139 | Derivative of pEGFP-C1 encoding a fusion of EGFP to CT288 (from L2/434 strain) without its amino acid residues from position 1 to 88 and from position 242 to 291 (EGFP-$CT288_{\Delta N\Delta TMD}$). Nucleotides 265 to 723, and 874 to 1689 of *ct288* were amplified by PCR from L2/434 chromosomal DNA using primers #944 and #863, and #862 and #954, respectively. The DNA products were then fused by overlapping PCR using primers #944 and #954. The final DNA product was digested with KpnI-BamHI and ligated into those sites of pEGFP-C1. | This work. |

**Table S1.** ***Continued.***

| Plasmid | Description/Construction | Reference |
|---|---|---|
| pFA147 | Y2H plasmid encoding a fusion of Gal4BD-*myc* to CT288 (from L2/434 strain) without its amino acid residues from position 1 to 88 and from position 242 to 291 (Gal4BD-*myc*-CT288$_{\Delta N\Delta TMD}$). Nucleotides 265 to 723, and 874 to 1689, of *ct288* were amplified by PCR from L2/434 chromosomal DNA using primers #944 and #863, and #862 and #954, respectively. The DNA products were then fused by overlapping PCR using primers #944 and #954. The final DNA product was digested with NdeI-BamHI and ligated into those sites of pGBKT7. | This work. |
| pFA164 | Derivative of pEGFP-C1 encoding a fusion of EGFP to full-length CCDC146 (EGFP-CCDC146$_{FL}$). Nucleotides 1 to 2865 of *CCDC146* were amplified by PCR from DNA of pCCDC146 using primers #1350 and #1351. The DNA product was digested with XhoI-EcoRI and ligated into those sites of pEGFP-C1. | This work. |
| pFA167 | Derivative of pEF6/*myc*-His C encoding full-length (CCDC146$_{FL}$) with a C-terminal HA epitope tag (CCDC146$_{FL}$-2HA). Nucleotides 1 to 865 of *CCDC146* were amplified by PCR from pCCDC146 using primers #1352 and #1355. The DNA product was digested with EcoRI-NotI and ligated into those sites of pEF6/*myc*-His C. | This work. |

**Table S1.** ***Continued.***

| Plasmid | Description/Construction | Reference |
|---|---|---|
| pFA168 | Derivative of pEF6/*myc*-His C encoding the C-terminal region (amino acid residues 692 to 955) of CCDC146 with C-terminal HA epitope tag (CCDC146$_{692-955}$-HA). Nucleotides 2074 to 2865 of CCDC146 were amplified by PCR from pCCDC146 DNA using primers #1354 and #1355. The DNA product was digested with EcoRI-NotI and ligated into those sites of pEF6/*myc*-His C. | This work. |
| pFA178 | Y2H plasmid encoding a fusion of Gal4BD-*myc* to the central region (amino acid residues 89 to 241) of CT288 (Gal4BD-*myc*-CT288$_{89-241}$). Nucleotides 265 to 723 of *ct288* were amplified by PCR from L2/434 chromosomal DNA using primers #944 and #1474. The DNA product was digested with NdeI-BamHI and ligated into those sites of pGBKT7. | This work. |
| pFA179 | Y2H plasmid encoding a fusion of Gal4BD-*myc* to the C-terminal region (amino acids residues 292 to 563) of CT288 (Gal4BD-*myc*-CT288$_{292-563}$). Nucleotides 874 to 1689 of *ct288* were amplified by PCR from L2/434 chromosomal DNA using primers #1475 and #954. The DNA product was digested with NdeI-BamHI and ligated into those sites of pGBKT7. | This work. |

**Table S1.** ***Continued.***

| **Plasmid** | **Description/Construction** | **Reference** |
|---|---|---|
| pFA181 | Y2H plasmid encoding a fusion of Gal4AD-HA to the N-terminal region (amino acid residues 1 to 691) of CCDC146 (Gal4AD-HA-CCDC146$_{1\text{-}691}$). Nucleotides 1 to 2073 of *ccdc146* were amplified by PCR from pCCDC146 DNA using primers #1476 and #1593. The DNA product was digested with NdeI-BamHI and ligated into those sites of pGADT7. | This work. |
| pFA184 | Y2H plasmid encoding a fusion of GalBD-*myc* to CT288 (from the C/TW3 strain) without its amino acid residues from position 1 to 88 and from position 242 to 291 (Gal4BD-*myc*-CT288$_{\Delta N\Delta TMD}$). Nucleotides 265 to 723, and 874 to 1689, of *ct288* were amplified by PCR from C/TW3 chromosomal DNA using primers #944 and #863, and #862 and #954, respectively. The construction of the plasmid was then done as for pFA147. | This work. |
| pFA185 | Y2H plasmid encoding a fusion of GalBD-*myc* to CT288 (from the E/Bour strain) without its amino acid residues from position 1 to 88 and from position 242 to 291 (Gal4BD-*myc*-CT288$_{\Delta N\Delta TMD}$). Nucleotides 265 to 723, and 874 to 1689, of *ct288* were amplified by PCR from C/TW3 chromosomal DNA using primers #944 and #863, and #862 and #954, respectively. The construction of the plasmid was then done as for pFA147. | This work. |

**Table S1.** ***Continued.***

| Plasmid | Description/Construction | Reference |
|---|---|---|
| pFA195 | Y2H plasmid encoding a fusion of Gal4AD-HA to full-length CCDC146 (Gal4AD-HA-CCDC146$_{FL}$). Nucleotides 1 to 2865 of *ccdc146* were amplified by PCR from pCCDC146 DNA using primers #1476 and #1707. The DNA product was digested with NdeI-EcoRI and ligated into those sites of pGADT7. | |
| pFA196 | Derivative of pEGFP-C1 encoding a fusion of EGFP to the C-terminal region (amino acid residues 692 to 955) of CCDC146 (EGFP-CCDC146$_{692\text{-}955}$). Nucleotides 2074 to 2865 of *CCDC146* were amplified by PCR from pCCDC146 DNA using primers #1730 and #1351. The DNA product was digested with XhoI-EcoRI and ligated into those sites of pEGFP-C1. | This work. |
| pFA197 | Derivative of pEF6/*myc*-His C encoding CT288 (from L2/434 strain) without its amino acid residues from position 1 to 88 and from position 242 to 291 and with a C-terminal HA epitope tag (CT288$_{\Delta N \Delta TMD}$-HA). Nucleotides 265 to 723, and 874 to 1689 of ct288 were amplified by PCR from pSVP255 DNA using primers #1733 and #863, and #862 and #1734, respectively. The DNA products were then fused by overlapping PCR using primers #1733 and #1734. The final DNA product was digested with BamHI-EcoRI and ligated into those sites of pEF6/*myc*-His C. | This work. |

**Table S1.** ***Continued.***

| Plasmid | Description/Construction | Reference |
|---|---|---|
| pML2 | Derivative of pDFTT3 aad carrying *ctl0560* from *C. trachomatis* strain L2/434 (ortologue of *ct288* in strain D/UW3) intron targeting sequence. For this, the 5' intron sequence was amplified by PCR using primers #2186, #2187, #2188, and #1922. The resulting ~350 bp DNA product was digested with HindIII and BsrG1/Bsp1407I and ligated into those sites of pDFTT3 aad. | This work. |
