# Supplementary material for: The Human Centrosomal Protein CCDC146 Binds Chlamydia trachomatis Inclusion Membrane Protein CT288 and Is Recruited to the Periphery of the Chlamydia-Containing Vacuole
Source: Front Cell Infect Microbiol. 2018 Jul 26;8:254. doi: 10.3389/fcimb.2018.00254 (PMC6070772; doi:10.3389/fcimb.2018.00254)
Supplement: Table S2 — DNA primers used in this work. [file Table_2.PDF]

Table S2. DNA primers used in this work.

Code	Description	Sequence	Restriction enzyme[a]
573	Complementary to *ct288*; PCR verification of strain *ct288::aadA*	GATCGGATCCCGTTTATTTTAGAGCTCATCAACC	-
599	Complementary to *ct288*; DNA sequencing.	AGTTCTTGGTTCCAGATACG	-
600	Complementary to *ct288*; DNA sequencing.	TTCTGAGTCTAGAAGCTGCG	-
627	Complementary to pEGFP-C1; DNA sequencing	TGGTCCTGCTGGAGTTCGTG	-
628	Complementary to pEGFP-C1; DNA sequencing	TTATGTTTCAGGTTCAGGG	-
642	Complementary to pEF6/*myc*-His C; DNA sequencing	TAATACGACTCACTATAGGGC	-
644	Complementary to pGADT7; DNA sequencing	CTATTCGATGATGAAGATACCCCACCAAACC	-
645	Complementary to pGADT7; DNA sequencing	AGTGAACTTGCGGGGTTTTTCAGTATCTACGA	-
862	Construction of pFA147, pFA184, pFA185, pFA139, and pFA197; locus-specific DNA sequencing of strain *ct288::aadA*	GCCAGCCGCACACTATATAAGTATTTGCTCGAACATTCTCC	-
863	Construction of pFA147, pFA184, pFA185, pFA139, and pFA197	GGAGAATGTTCGAGCAAATACTTATATAGTGTGCGGCTGGC	-
944	Construction of pFA147, pFA178, pFA184, pFA185, and pFA139	GGAATTCCATATGAAAAAGGCTCTGGCTCAACG	NdeI
954	Construction of pFA147, pFA179, pFA184, pFA185, and pFA139	CGCGGATCCTTAGTGATTATCTAACAGG	BamHI
977	Complementary to pGBKT7; DNA sequencing	AAATCATAAGAAATTCGC	-
1350	Construction of pFA164	GATCCTCGAGCTGAAGACAGTAGCACAGACAC	XhoI
1351	Construction of pFA164 and pFA196	GATCGAATTCTCAGATTTCAACTGGCTTTATAACAGG	EcoRI
1352	Construction of pFA167	GATCGAATTCACCATGGAAGACAGTAGCACAGACAC	EcoRI
1354	Construction of pFA168	GATCGAATTCACCATGGAAATTTGTGTGACCCAG	EcoRI

Table S2. ***Continued.***

Code	Description	Sequence	Restriction enzyme[a]
1355	Construction of pFA167 and pFA168	ATAAGAATGCGGCCGCTTAAGCATAATCAGGAACATCATACGGATAGATTTCAACTGGCTTTATAACAGG	NotI
1356	Complementary to *CCDC146*; DNA sequencing	TAAGAAAGATGGAACTGC	-
1357	Complementary to *CCDC146*; DNA sequencing	TCTACTAACTTAGACTCC	-
1358	Complementary to *CCDC146*; DNA sequencing	ATGAAATCCTTCTCCAGC	-
1474	Construction of pFA178 and PCR verification of strain *ct288::aadA*	CGCGGATCCTTAATATAGTGTGCGGCTGGC	BamHI
1475	Construction of pFA179	GGAATTCCATATGAAGTATTTGCTCGAACATTCTCC	NdeI
1476	Construction of pFA181 and pFA195	GGAATTCCATATGGAAGACAGTAGCACAGACAC	NdeI
1546	Construction of pSVP255	GATCGGTACCAACGGAGCCTTCTAGCTATTTTG	KpnI
1565	Construction of pSVP255	ATCTGTCGAAGTGAGGTTTATGGTTTATTTTAGAGCTCATC	-
1566	Construction of pSVP255	GATGAGCTCTAAAATAAACCATAAACCTCACTTCGACAGAT	-
1567	Construction of pSVP255	GATCGCGGCCGCGGTGATTATCTAACAGGTATTG	NotI
1593	Construction of pFA181	CGCGGATCCTTATCTTTGCTTCTCAGCAATCTTC	BamHI
1707	Construction of pFA195	GATCGAATTCTCAGATTTCAACTGGCTTTATAACAGG	EcoRI
1730	Construction of pFA196	GATCCTCGAGCTCAAATTTGTGTGACCCAG	XhoI
1733	Construction of pFA197	GATCGGATCCACCATGGCGAAGGCTCTGGCTCAAGC	BamHI
1734	Construction of pFA197	GATCGAATTCTTAAGCATAATCAGGAACATCATACGG	EcoRI
1864	Complementary to group II intron; PCR verification of strain *ct288::aadA*	ACGGATGCCGAGAATCTG	-
1865	Complementary to group II intron; PCR verification of strain *ct288::aadA*	TCTCGGAGTATACGGCTCTG	-
1898	locus-specific DNA sequencing of strain *ct288::aadA*	GATCGAATTCATATAGTGTGCGGCTGGCATG	-

Table S2. ***Continued.***

**Code**	**Description**	**Sequence**	**Restriction enzyme**[a]
1922	Group II intron retargeting (EBS universal); locus-specific DNA sequencing of strain *ct288::aadA*	CGAAATTAGAAACTTGCGTTCAGTAAAC	-
2186	Group II intron retargeting (288_114|115s_IBS1/2); locus-specific DNA sequencing of strain *ct288::aadA*	AAAAAAGCTTATAATTATCCTTAATGTGCATTACGGTGCGCCCAGATAGGGTG	HindIII
2187	Group II intron retargeting (288_114|115s_EBS1/d)	CAGATTGTACAAATGTGGTGATAACAGATAAGTCATTACGGGTAACTTACCTTTCTTTGT	BsrG1/ Bsp1407I
2188	Group II intron retargeting (288_114|115s_EBS2); locus-specific DNA sequencing of strain *ct288::aadA*	TGAACGCAAGTTTCTAATTTCGGTTCACATCCGATAGAGGAAAGTGTCT	-
-	T7 promoter primer; DNA sequencing of pML2	TAATACGACTCACTATAGGG	

[a]Restriction sites are underlined.
