# Supplementary material for: The Human Centrosomal Protein CCDC146 Binds Chlamydia trachomatis Inclusion Membrane Protein CT288 and Is Recruited to the Periphery of the Chlamydia-Containing Vacuole
Source: Front Cell Infect Microbiol. 2018 Jul 26;8:254. doi: 10.3389/fcimb.2018.00254 (PMC6070772; doi:10.3389/fcimb.2018.00254)
Supplement: Table S3 — Candidate binding partners of C. trachomatis Inc protein CT288 identified in a yeast two-hybrid screen using a HeLa cDNA library as bait. [file Table_3.PDF]

Table S3. Candidate binding partners of *C. trachomatis* Inc protein CT288 identified in a yeast two-hybrid screen using a HeLa cDNA library as bait.

**Candidate interacting partner / Accession**	**Protein fragment (amino acids; aa)**	**Number of hits**
Coiled-coil domain-containing protein 146 (CCDC146) / NP_065930.2	692-955 (out of 955 aa)	6 (out of 29)
Coiled-coil domain-containing protein 146 (CCDC146) / NP_065930.2	721-955 (out of 955 aa)	3 (out of 29)
Leber-congenital amaurosis 5 like protein (LCA5L) / NP_689718.1	5-279 (out of 670 aa)	2 (out of 29
Anterior gradient 3 homolog (*Xenopus laevis*) (AGR3) / AAH58284.1	1-166 (out of 166 aa)	1 (out of 29)
Coiled-coil domain-containing protein 42 (CCDC42) / NP_001151733.1	1-242 (out of 242 aa)	1 (out of 29)
Inhibitor of kappa light polypeptide gene enhancer in B-cells (IKBKAP) / EAW59030.1	1156-1331 (out of 1452 aa)	1 (out of 29)
Fasciculation and elongation protein zeta 2 (zygin II) (FEZ2) / EAX00424.1	181-353 (out of 353 aa)	1 (out of 29)
THO complex 7 homolog (*Drosophila*) (THOC7) / AAH65012.1	1-204 (out of 204 aa)	1 (out of 29)
Exocyst complex component 1 (EXOC1) / AAH94751.1	120-358 (out of 894 aa)	1 (out of 29)
MLF1 interacting protein (CENP-50) / AAI31557.1	194-418 (out of 418 aa)	1 (out of 29)
Catenin (cadherin-associated protein) / NP_001158355.1	690-860 (out of 860 aa)	1 (out of 29)
COP9 constitutive photomorphogenic homolog subunit 4 (*Arabidopsis*) / EAX05922.1	1-406 (out of 406 aa)	1 (out of 29)
Protein phosphatase 2, regulatory subunit B' (PP2A) / AAC37601.1	331-486 (out of 486)	1 (out of 29)
Structural maintenance of chromosomes 3 (SMC3) / AAH47324.1	828-1217 (out of 1217 aa)	1 (out of 29)
Superkiller viralicidic activity 2-like (*S. cerevisiae*) / EAX03557.1	895-1042 (out of 1042 aa)	1 (out of 29)
Interferon-induced protein 44-like (IFI44L) / NP_006811.2	327-452 (out of 452 aa)	1 (out of 29)
Mediator complex subunit 4 / AAH05189.1	1-270 (out of 270 aa)	1 (out of 29)
Fasciculation and elongation protein zeta 1 (zygin I) / Q99689.1	238-392 (out of 392 aa)	1 (out of 29)
Inhibitor of growth family, member 5 (ING5) / AAH71899.1	1-130 (out 240 aa)	1 (out of 29)
Nucleoporin 88kDa (NUP88) / AAH00335.1	517-741 (out of 741 aa)	1 (out of 29)
Furry homolog (*Drosophila*) (FRY) / NP_055845.1	2782-3013 (out of 3013 aa)	1 (out of 29)
